# LncRNA PVT1 Knockdown Ameliorates Myocardial Ischemia Reperfusion Damage via Suppressing Gasdermin D-Mediated Pyroptosis in Cardiomyocytes

**DOI:** 10.3389/fcvm.2021.747802

**Published:** 2021-09-14

**Authors:** Cuizhi Li, Huafeng Song, Chunlin Chen, Shaoxian Chen, Qiyu Zhang, Dehui Liu, Jinglong Li, Haojian Dong, Yueheng Wu, Youbin Liu

**Affiliations:** ^1^Department of Cardiology, Guangzhou Eighth People's Hospital, Guangzhou Medical University, Guangzhou, China; ^2^Guangdong Provincial Key Laboratory of South China Structural Heart Disease, Guangdong Cardiovascular Institute, Guangdong Provincial People's Hospital and Guangdong Academy of Medical Sciences, School of Medicine, South China University of Technology, Guangzhou, China; ^3^Department of Cardiology, Guangdong Provincial People's Hospital, Guangzhou, China

**Keywords:** lncRNA, PVT1, myocardial ischemia reperfusion, pyroptosis, gasdermin D

## Abstract

**Objective:** Myocardial ischemia reperfusion (I/R) damage is a life-threatening vascular emergency after myocardial infarction. Here, we observed the cardioprotective effect of long non-coding RNA (lncRNA) PVT1 knockdown against myocardial I/R damage.

**Methods:** This study constructed a myocardial I/R-induced mouse model and a hypoxia/reoxygenation (H/R)-treated H9C2 cells. PVT1 expression was examined via RT-qPCR. After silencing PVT1 via shRNA against PVT1, H&E, and Masson staining was performed to observe myocardial I/R damage. Indicators of myocardial injury including cTnI, LDH, BNP, and CK-MB were examined by ELISA. Inflammatory factors (TNF-α, IL-1β, and IL-6), Gasdermin D (GSDMD), and Caspase1 were detected via RT-qPCR, western blot, immunohistochemistry, or immunofluorescence. Furthermore, CCK-8 and flow cytometry were presented for detecting cell viability and apoptosis.

**Results:** LncRNA PVT1 was markedly up-regulated in myocardial I/R tissue specimens as well as H/R-induced H9C2 cells. Silencing PVT1 significantly lowered serum levels of cTnI, LDH, BNP, and CK-MB in myocardial I/R mice. H&E and Masson staining showed that silencing PVT1 alleviated myocardial I/R injury. PVT1 knockdown significantly lowered the production and release of inflammatory factors as well as inhibited the expression of GSDMD-N and Caspase1 in myocardial I/R tissue specimens as well as H/R-induced H9C2 cells. Moreover, silencing PVT1 facilitated cell viability and induced apoptosis of H/R-treated H9C2 cells.

**Conclusion:** Our findings demonstrated that silencing PVT1 could alleviate myocardial I/R damage through suppressing GSDMD-mediated pyroptosis *in vivo* and *in vitro*. Thus, PVT1 knockdown may offer an alternative therapeutic strategy against myocardial I/R damage.

## Introduction

Myocardial ischemia reperfusion (I/R) may trigger acute myocardial infarction, with increasing morbidity and morbidity in modern society and trending to be younger (1–3). Myocardial I/R represents an intricate pathological process in which the blood supply is restored following myocardial ischemia, leading to metabolic dysfunction as well as structural injury (4–6). The potential mechanism of myocardial I/R is of complexity, involving systematic networks (7). Thus, mitigating the injury mediated by myocardial I/R has attracted scholars' attention of globally.

Pyroptosis, a type of inflammatory cell deaths, has the characteristics of gasdermin D (GSDMD) or gasdermin E (GSDME)-mediated necrosis, activation of proinflammatory caspase-1 and excessive release of inflammatory factors [such as interleukin 1β (IL-1β), interleukin 6 (IL-6), and tumor necrosis factor α (TNF-α)] (8). GSDMD-mediated pyroptosis of cardiomyocytes represents a critical event for myocardial I/R damage and caspase-11/GSDMD signaling is essential for this process (9). Suppressing GSDMD distinctly alleviates I/R-mediated myocardial damage via reducing pyroptosis of cardiomyocytes (10). Long non-coding RNA (LncRNA) is a class of non-coding RNA with >200 nt length (11). Growing evidence demonstrates that lncRNAs exert key roles in myocardial I/R via mediating different biological processes including pyroptosis (12). For instance, lncRNA H19 may initiate microglial pyroptosis as well as neuronal deaths in retinal I/R damage (13). LncRNA KLF3-AS1 may ameliorate cardiomyocyte pyroptosis and myocardial infarction (14). LncRNA GAS5 induces pyroptosis of cardiac fibroblasts through mediating NOD-like receptor protein 3 (NLRP3) (15). LncRNA MEG3 facilitates cerebral I/R injury via enhancing pyroptosis (16). Nevertheless, the influence of lncRNAs in regulating pyroptosis in cardiovascular diseases especially myocardial I/R damage is mostly unclear. Previous research has reported that lncRNA plasmacytoma variant translocation 1 (PVT1) up-regulation is involved in pathogenesis of cardiovascular diseases (17). Knockdown of PVT1 suppresses apoptosis of vascular smooth muscle cells as well as extracellular matrix destruction for abdominal aortic aneurysms (18). Inhibiting PVT1 alleviates atrial fibrosis and atrial fibrillation (19). Serum PVT1 levels are distinctly up-regulated in coronary artery disease and distinguish mild and severe patients (20). Nevertheless, it remains unknown about the influence and mechanisms of PVT1 on myocardial I/R. Here, our study found that lncRNA PVT1 was markedly up-regulated in myocardial I/R mouse model and hypoxia/reoxygenation (H/R)-induced cellular model. Knockdown of PVT1 alleviated myocardial I/R damage via suppressing GSDMD-mediated pyroptosis in cardiomyocytes.

## Materials and Methods

### Animals and Grouping

Totally, 50 C57BL/6 mice (Nanjing Junke Biological Engineering Co., Ltd.) were fed in a specific pathogen-free environment under a 12 h light/12 h dark cycle. Each animal experiment strictly followed the Guide for the Care and Use of Laboratory Animal by International Committees. The mice were randomly separated into the following five groups before surgery (10 mice/group): control group; sham operation group; I/R group; I/R + short hairpin RNA (shRNA) against negative control (sh-NC) group; I/R + shRNA against PVT1 (sh-PVT1) group. This study gained the approval of the Institutional Animal Care of Guangdong Provincial People's Hospital (GDREC2016255H).

### Preparation of I/R Animal Model

After the mice were adaptively reared for 1 week, they were anesthetized by intraperitoneal injection of sodium pentobarbital (45 mg/kg). After anesthesia, there was no response in the clip toe test and the trachea was intubated. The ventilator parameters were set to synchronize with the respiratory rate of the anesthetized mice. Then, the endotracheal tube was connected to the ventilator. Before the operation, a physiological signal collection and processing system was used to record the rat's electrocardiogram. The chest was opened through the third intercostal space on the left edge of the sternum, and the left anterior descending (LAD) coronary artery was ligated with 7-0 silk thread. After 45 min, the ligature was loosened for reperfusion. No treatment was done for mice in the control group. This study only opened the chest of mice in the sham operation group, but threaded the corresponding part without ligating the LAD. After the operation, mice were continuously monitored by ECG for 15 min and placed on a heating pad at 30°C until they were awakened. 48 h before modeling, mice in the I/R + sh-NC and I/R + sh-PVT1 groups were injected by 10 μL sh-NC or sh-PVT1 (GenePharma, Shanghai, China) that was premixed with Lipofectamine 2000 reagent (Thermo Fisher Scientific, USA). The sequence of sh-PVT1 was as follows: 5′-CCUGCAUAACUAUCUGCUUTT-3′.

### Sample Collection and Preparation

Under anesthesia, abdominal aortic blood was firstly collected. Then, the limbs were fixed, the chest was opened and the mouse's heart was obtained. After removing the auricle and vascular tissue, the heart was transected into two parts. Half of the tissue at the bottom of the heart was quick-frozen by liquid nitrogen, and then transferred to a −80°C refrigerator for subsequent experiments. Apical 1/2 tissue was fixed in 40 g/L paraformaldehyde and transferred to 70% ethanol solution on the second day. Then, through the Excelsior™ AS tissue processing system, the tissue was dehydrated by gradient ethanol, transparent by xylene and waxed in liquid paraffin. Subsequently, the HistoCore Arcadia paraffin embedding system was used for pathological examination after embedding and retention.

### Real-Time Quantitative Reverse Transcription PCR (RT-qPCR)

Total RNA was extracted from myocardial tissues and cells utilizing TRIzol (Beyotime, Shanghai, China) and cDNA was reverse transcribed with PrimeScript RT Master Mix (Servicebio, Wuhan, China). These primer sequences were as follows: PVT1: 5′-TGAGAACTGTCCTTACGTGACC-3′ (F), 5′-AGAGCACCAAGACTGGCTCT-3′ (R); IL-1β: 5′-CACCTCTCAAGCAGAGCACAG-3′ (F), 5′-GGGTTCCATGGTGAAGTCAAC-3′ (R); IL-6: 5′-GCTACAGCACAAAGCACCTG-3′ (F), 5′-GACTTCAGATTGGCGAGGAG-3′ (R); TNF-α: 5′-GGCAGCCTTGTCCCTTGAAGAG-3′ (F), 5′-GTAGCCCACGTCGTAGCAAACC-3′ (R); GSDMD: 5′-CCAACATCTCAGGGCCCCAT-3′ (F), 5′-TGGCAAGTTTCTGCCCTGGA-3′ (R); GAPDH: 5′-CTGGGCTACACTGAGCACC-3′ (F), 5′-AAGTGGTCGTTGAGGGCAATG-3′ (R). The expression of above targets was quantified with the 2^−ΔΔCt^ method, while GAPDH served as an internal control.

### Enzyme-Linked Immunosorbent Assay (ELISA)

Abdominal aortic blood samples were centrifuged at 3,000 g. Then, the serum samples were harvested. Following the instructions of ELISA kits, brain natriuretic peptide (BNP; Biocompare, USA), cardiac troponin I (cTnI; Biocompare, USA) and IL-1β (Biocompare, USA) levels were tested in serum or cell supernatant. Through automatic biochemical analyzer, lactate dehydrogenase (LDH) and creative kinase isoenzyme MB (CK-MB) levels were examined in serum or cell supernatant.

### Histological Analysis

The paraffin-embedded mouse myocardial tissue was sectioned to 5 μm. H&E staining and Masson staining were carried out following the kit instructions (Servicebio, Wuhan, China). The paraffin sections were deparaffinized to water. Then, H&E staining was successively performed. The sections were stained with hematoxylin for 5 min and rinsed by tap water, followed by being differentiated by differentiation solution for 3 s. After being rinsed by tap water, the sections returned to blue for about 3 s, rinsed by tap water and dehydrated by 85 and 95% ethanol in turn for 4 min. Afterwards, the sections were stained by eosin dye solution for 5 min and dehydrated with anhydrous ethanol for 3 times (5 min each time) and transparent by xylene twice for 2 min. Then, the sections were mounted with neutral gum. Masson staining was carried out according to the following procedures. The sections were stained through Masson A solution lasting 15 h as well as heated in a 65°C oven lasting 30 min. After being washed using tap water, the sections were stained by Masson B solution and C solution mixed dyeing for 1 min and differentiated with 1% hydrochloric acid alcohol lasting 1 min. Then, the sections were washed by tap water as well as stained utilizing Masson D solution lasting 6 min and immersed by Masson E solution for 1 min and Masson F solution for 15 s. Afterwards, the sections were differentiated by 1% glacial acetic acid for 3 times (8 s each time) and dehydrated with absolute ethanol for 3 times (5 min each time). After being transparent, the slides were mounted with neutral gum. All slides were scanned at 200 × by TissueFAXS PLUS scanning system (Taborstrasse, Austria).

### Western Blot

According to the instructions of the reagents, protein specimens were extracted from myocardial tissues, serum samples and cells through RIPA lysate. After centrifugation at 14,000 g, the supernatant was collected and mixed with the loading buffer. The protein was denatured by boiling at 100°C for 10 min. About 50 μg protein was electrophoresed in 10% SDS-PAGE (Beyotime, Shanghai, China), then transferred to 0.22 μm PVDF membrane (Merck KGaA, Germany). Afterwards, the membrane was incubated with primary antibodies against α-myosin heavy chain (α-MHC; 1/1,000; ab174640; Abcam, USA), β-myosin heavy chain (β-MHC; 1/1,000; ab170867; Abcam, USA), TNF-α (1/1,000; ab215188; Abcam, USA), IL-1β (1/1,000; ab254360; Abcam, USA), IL-6 (1/1,000; ab259341; Abcam, USA), GSDMD (1/200; ab219800; Abcam, USA), TLR4 (1/50; ab1355; Abcam, USA), MyD88 (1/1,000; ab219413; Abcam, USA), NLRP3 (1/1,000; ab263899; Abcam, USA), NF-κB phosphorylation p-p65 (1/1,000; ab76302, USA), NF-κB p65 (#8242, 1:1,000; Cell Signaling Technology, USA), Cleaved caspase-3 (1/5,000; ab21443; Abcam, USA), Bax (1/1,000; ab23247; Abcam, USA), Bcl-2 (1/1,000; ab259833; Abcam, USA), Caspase1 (1/1,000; ab207802; Abcam, USA), and GAPDH (1/1,000; ab9484; Abcam, USA) overnight at 4°C. The next day, the membrane was washed utilizing TBST as well as incubated with HRP-conjugated secondary antibodies (1/10,000; ab7090; Abcam, USA) lasting 1 h at room temperature. Then, the ECL substrate (Thermo Scientific, USA) was dropped on the membrane, followed by being exposed in the FluorChem™E chemiluminescence imaging system. The integrated optical density of the protein band was measured using ImageJ software (version 1.8.0).

### Immunohistochemistry

Paraffin-embedded sections of myocardial tissue were baked in a 60°C incubator for 2 h. The sections were deparaffinized by xylene and hydrated with ethanol gradient. 3% hydrogen peroxide was added to eliminate endogenous peroxidase activity. After washing, antigen retrieval was carried out in PBS buffer. Then, the sections were blocked by goat serum for 15 min. The primary antibody against GSDMD (1/200; sc-393581; Santa Cruz Biotechnology, USA) and Caspase1 (1/1,000; ab207802; Abcam, USA) was added dropwise to the sections and incubated overnight at 4°C. After washing with PBS, the sections were incubated with secondary antibody (1/10,000; ab7090; Abcam, USA) lasting 2 h. Then, the color was developed with DAB color developing solution. The sections were counterstained by hematoxylin, dehydrated, transparent, and mounted. The results were investigated under a microscope, which were analyzed by Image Plus image analysis software.

### Cell Culture, Treatment, and Transfection

H9C2 cells (ATCC, USA) were grown in DMEM cell culture medium containing 1.5 g/L NaHCO_3_, 10% FBS, 1% glutamine, and 1% penicillin in a constant temperature incubator of 5% CO_2_ at 37°C. For H/R, the cells were cultured in an environment of 95% N_2_ and 5% CO_2_ at 37°C lasting 4 h. After changing the medium, the cells were reoxygenated with 5% CO_2_ at 37°C lasting 3 h. PVT1 sequence was designed and synthesized by GenePharma company (Shanghai, China), which was subcloned into pcDNA3.1 (Invitrogen, USA). The pcDNA3.1 vector served as a control. Lipofectamine 2000 (Invitrogen, USA) was utilized for transfection of plasmids (500 ng pcDNA3.1 vector (empty vector), 500 ng PVT1) into H9C2 cells for 48 h. The sense and antisense oligonucleotides of the sh-PVT1 (5′-CCUGCAUAACUAUCUGCUUTT-3′) were synthesized and cloned into the pENTR™/U6 vector (Invitrogen, USA). For silencing PVT1, H9C2 cells (5 × 10^4^) were transfected by 1 μg sh-NC or 1 μg sh-PVT1 for 48 h via Lipofectamine 2000 reagent before H/R. After transfection for 48 h, above transfected cells were used for the subsequent experiments. Furthermore, the cells were treated with 25 nM Necrosulfonamide (NSA; abs814352; Absin, Shanghai, China), followed by PVT1 overexpression vector transfection and H/R treatment.

### Cell Counting Kit-8 (CCK-8)

H9C2 cells in each group were planted onto a 96-well plate (1 × 10^4^/well). Each group set eight multiple holes. A blank group was set, which was only added by culture medium without cells. Each well was incubated with 10 μL of CCK-8 solution (Dojindo, Japan) lasting 1 h at 37°C. After incubation, a microplate reader was utilized for measuring the absorbance at 450 nm wavelength.

### Flow Cytometry for Apoptosis Detection

Annexin V-fluorescein isothiocyanate (FITC)/propidium iodide (PI) cell apoptosis kit (Beyotime, Shanghai, China) was utilized for detecting cellular apoptosis. H9C2 cells were collected and washed utilizing PBS. The cells were resuspended by 250 μL prepared binding buffer. The density of the cells was adjusted to 1 × 10^9^/L. Then, 100 μL cell suspension was inoculated into a 5 mL flow tube. The cells were incubated with 5 μL Annexin V/FITC and 10 μL PI in the dark at room temperature. After 15 min, the apoptosis rate was detected by flow cytometry.

### Immunofluorescence

H9C2 cells were seeded and climbed on the 6-well plate. When the cell fusion was about 80%, the cells were treated with H/R, transfection and drug treatment. Then, the cells were fixed by paraformaldehyde, permeabilized by PBS containing 0.1% TritonX-100, and blocked by BSA. Then, the sections were incubated with primary antibody against GSDMD (1/200; ab219800; Abcam, USA), followed by FITC-labeled fluorescent secondary antibody (1/200; ab7064; Abcam, USA). After the nucleus was stained with 1 μg/ml DAPI for 10 min, images were investigated under a confocal laser microscope.

### Statistical Analysis

All analyses were presented utilizing Graphpad Prism software (version 7.0). Statistical differences between multiple groups were compared through one-way analysis of variance (ANOVA) followed by Tukey's test. The results were displayed as the mean ± standard deviation. Each experiment was carried out in triplicate. *P* < 0.05 was indicative of statistical significance.

## Results

### LncRNA PVT1 Is Up-Regulated in Myocardial I/R Tissues and Its Knockdown Alleviates Myocardial I/R Damage

**Figure 1:**
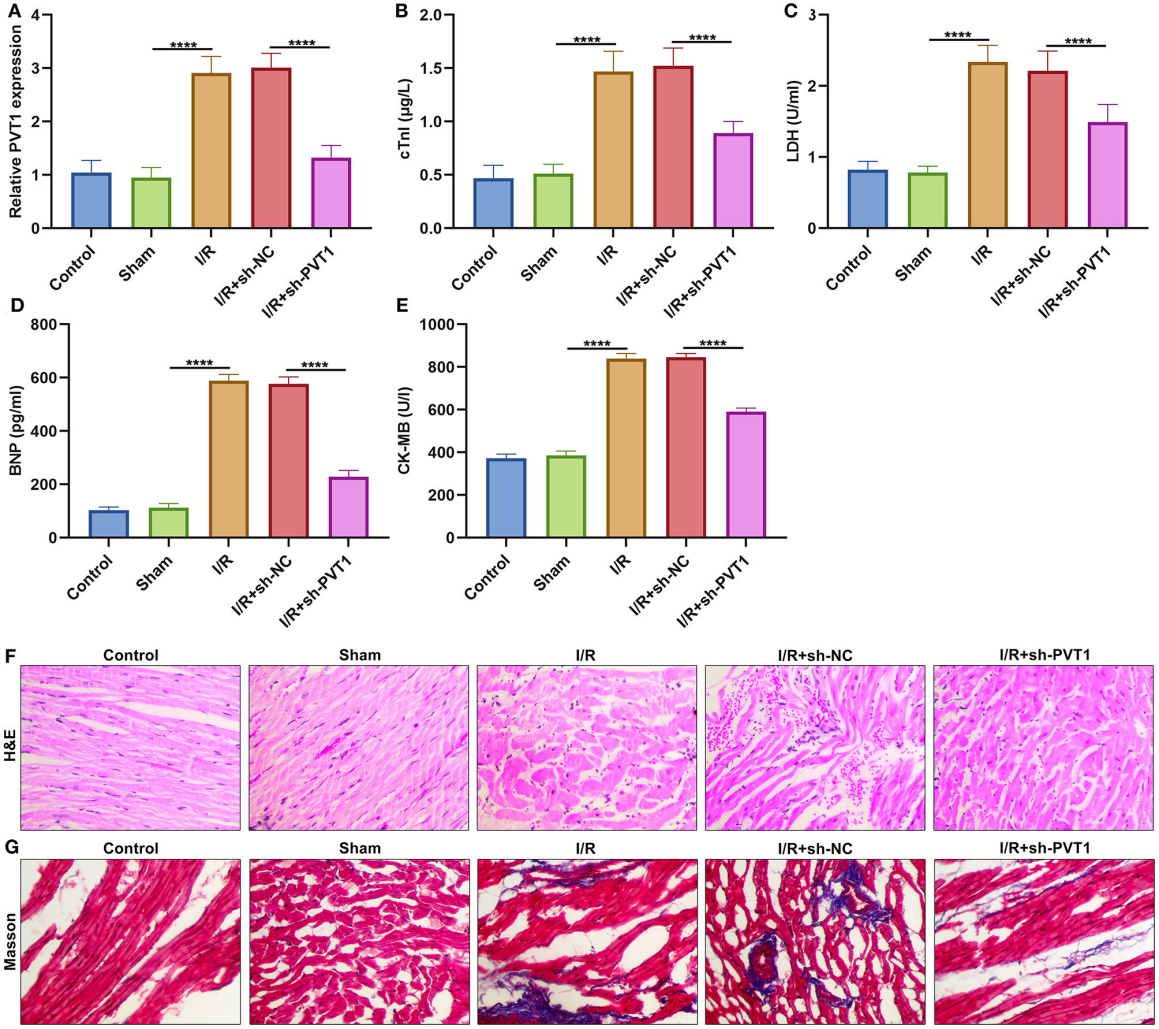
LncRNA PVT1 is up-regulated in myocardial I/R tissues and silencing PVT1 alleviates myocardial I/R damage. **(A)** RT-qPCR of the expression of lncRNA PVT1 in myocardial tissues in five groups: control, sham, I/R, I/R + sh-NC, and I/R + sh-PVT1 groups. Mice in the I/R + sh-NC and I/R + sh-PVT1 groups were injected by 10 μL sh-NC or sh-PVT1 via Lipofectamine 2000 reagent. After 48 h, myocardial I/R model was constructed. No treatment was done for mice in the control group. The chest of mice in the sham operation group was only opened, but was threaded the corresponding part without ligating the LAD. There were 10 mice in each group. **(B–E)** ELISA for examining the serum levels of **(B)** cTnI, **(C)** LDH, **(D)** BNP, and **(E)** CK-MB in above five groups. **(F)** HandE staining of myocardial tissues in five groups. Bar = 100 μm. **(G)** Masson staining of myocardial tissues in five groups. Bar = 100 μm. ****p < 0.0001.

Here, this study constructed a myocardial I/R mouse model. Our results showed that lncRNA PVT1 displayed significant up-regulation in myocardial I/R tissues compared with sham myocardial tissues (Figure 1A). This indicated that PVT1 might participate in myocardial I/R progression. For investigating this influence of PVT1 on myocardial I/R damage, PVT1 was silenced by its shRNA. In Figure 1A, the expression of PVT1 was markedly inhibited by sh-PVT1 in myocardial I/R tissues compared with sh-NC. Indicators of myocardial injury including cTnI, LDH, BNP and CK-MB were examined by ELISA. As demonstrated by our data, serum levels of cTnI (Figure 1B), LDH (Figure 1C), BNP (Figure 1D), and CK-MB (Figure 1E) were markedly elevated in myocardial I/R mice compared with sham mice. But PVT1 knockdown significantly decreased serum cTnI, LDH, BNP and CK-MB levels in myocardial I/R models. To evaluate the effect of PVT1 knockdown on the morphology of I/R heart tissue, H&E and Masson staining of heart tissue was performed at the end of the intervention. The results of H&E staining showed that the myocardial fibers of the control group and the sham group were arranged regularly; there was no breakage or necrotic gap; and the myocardial cell nucleus was fusiform or oval (Figure 1F). For I/R and I/R + sh-NC groups, the myocardial fiber structure was damaged; the myocardial fiber was broken and dissolved; the intermuscular space was enlarged; there were inflammatory cellular infiltrations in the infarct. The destruction of myocardial fiber structure and infiltrations of inflammatory cells were improved in I/R + sh-BCRT1 group. As shown in Masson staining, the myocardial tissue fibers were neatly arranged, evenly stained, and there was no collagen in the control group and the sham group (Figure 1G). For I/R and I/R + sh-NC groups, the degree of myocardial fibrosis was obvious; myocardial cells were significantly reduced; and there was increased collagen. For I/R + sh-BCRT1 mice, myocardial fibrosis was markedly ameliorated; the reduction of myocardial cells was improved; and the collagen was decreased. These findings demonstrated that PVT1 knockdown could alleviate myocardial I/R injury.

### LncRNA PVT1 Knockdown Alleviates Cytokine Release in Myocardial I/R Mice

**Figure 2:**
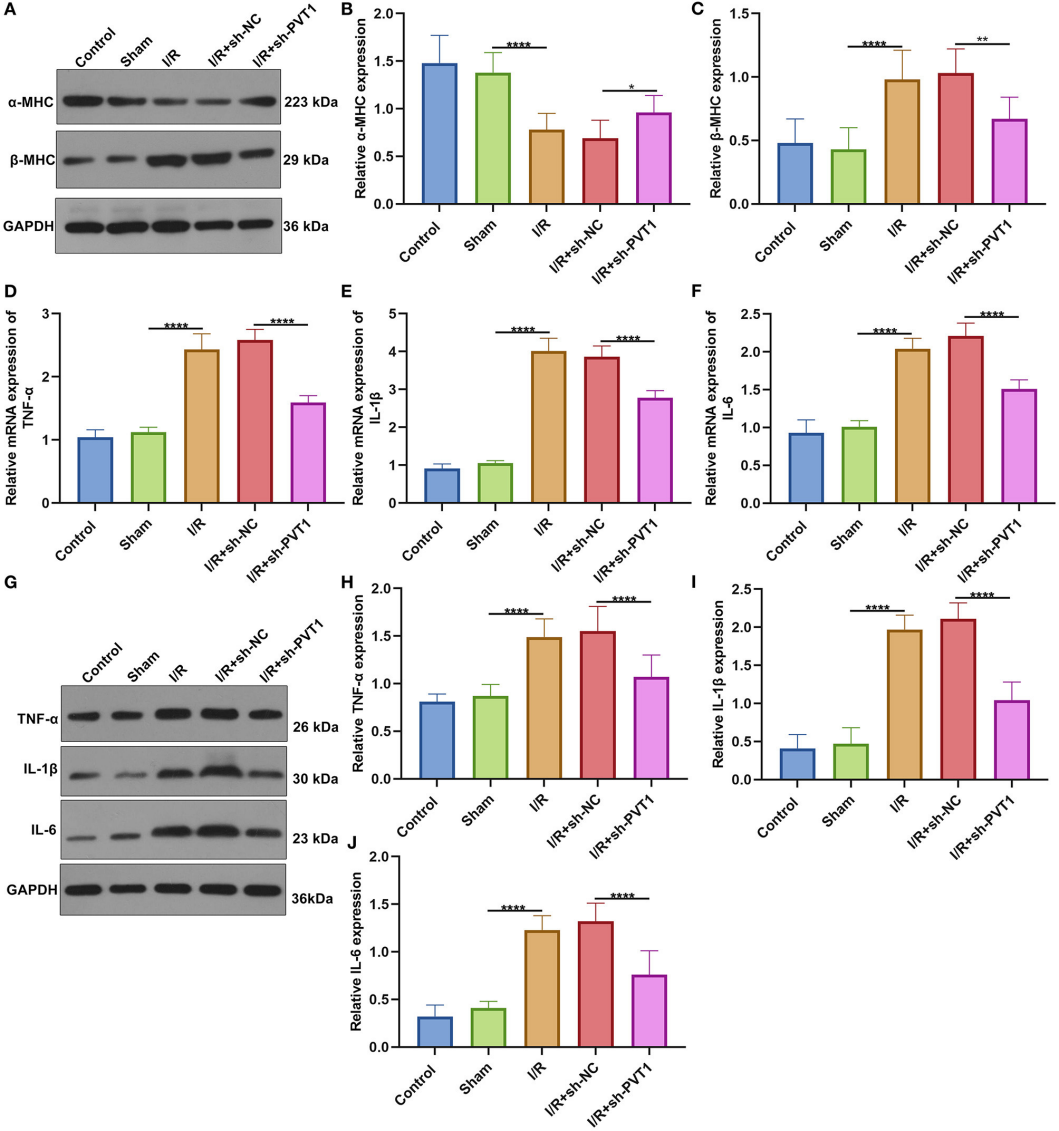
LncRNA PVT1 knockdown ameliorates cardiac function and cytokine release in myocardial I/R mice. **(A–C)** Western blot of the expression of **(B)** α-MHC and **(C)** β-MHC in myocardial tissues of control, sham, I/R, I/R + sh-NC, and I/R + sh-PVT1 groups. Mice in the I/R + sh-NC and I/R + sh-PVT1 groups were injected by 10 μL sh-NC or sh-PVT1 via Lipofectamine 2000 reagent. After 48 h, myocardial I/R model was constructed. No treatment was done for mice in the control group. The chest of mice in the sham operation group was only opened, but was threaded the corresponding part without ligating the LAD. There were 10 mice in each group. **(D–F)** RT-qPCR of the expression of **(D)** TNF-α, **(E)** IL-1β, and **(F)** IL-6 mRNAs in serum samples from above groups. **(G–J)** Western blot of the expression of **(H)** TNF-α, **(I)** IL-1β, and **(J)** IL-6 proteins in serum samples of each group. *p < 0.05; **p < 0.01; ****p < 0.0001.

Western blot was applied for testing the expression of cardiac function markers including α-MHC and β-MHC in myocardial tissues. Our data showed that α-MHC expression was significantly decreased while β-MHC expression was significantly increased in I/R myocardial tissues compared with sham tissues (Figures 2A–C). However, silencing PVT1 markedly alleviated I/R-induced the decrease in α-MHC and the increase in β-MHC. This indicated that PVT1 knockdown could improve cardiac function in myocardial I/R mice. Serum TNF-α, IL-1β, and IL-6 levels were examined by RT-qPCR. In comparison to sham mice, there were increased serum levels of TNF-α (Figure 2D), IL-1β (Figure 2E), and IL-6 (Figure 2F) mRNAs in myocardial I/R mice. Nevertheless, silencing PVT1 distinctly decreased their serum levels in myocardial I/R mice. Western blot was also carried out to test the expression of TNF-α, IL-1β, and IL-6 in serum samples (Figure 2G). Consistently, the expression TNF-α (Figure 2H), IL-1β (Figure 2I), and IL-6 (Figure 2J) proteins was markedly up-regulated in serum of myocardial I/R mice compared with sham mice. Their expression was significantly ameliorated by PVT1 knockdown. Therefore, silencing PVT1 could alleviate I/R-induced cytokine release.

### LncRNA PVT1 Knockdown Ameliorates GSDMD-Mediated Pyroptosis in Myocardial I/R Mice

**Figure 3:**
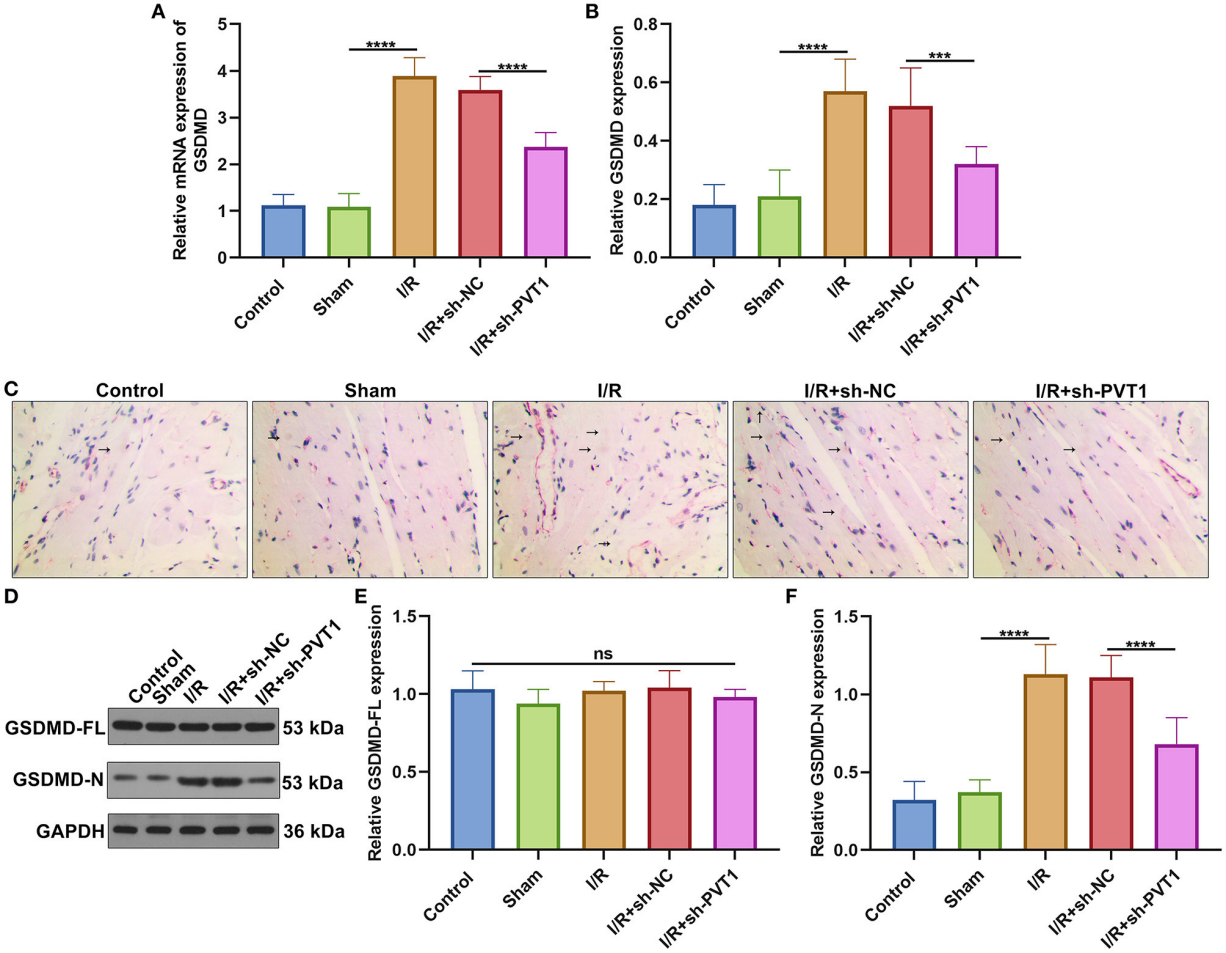
LncRNA PVT1 knockdown alleviates GSDMD-mediated pyroptosis in myocardial I/R mice. **(A)** RT-qPCR of the expression of GSDMD mRNA in myocardial tissues of five groups: control, sham, I/R, I/R + sh-NC, and I/R + sh-PVT1 groups. Mice in the I/R + sh-NC and I/R + sh-PVT1 groups were injected by 10 μL sh-NC or sh-PVT1 via Lipofectamine 2000 reagent. After 48 h, myocardial I/R model was constructed. No treatment was done for mice in the control group. The chest of mice in the sham operation group was only opened, but was threaded the corresponding part without ligating the LAD. There were 10 mice in each group. **(B,C)** Immunohistochemistry of the expression of GSDMD protein in myocardial tissues of above groups. Arrow highlights the positive staining of GSDMD. **(D–F)** Western blot of **(E)** GSDMD-FL and **(F)** GSDMD-N expressions in myocardial tissues of each group. Ns: not significant; ***p < 0.001; ****p < 0.0001.

Here, we detected the expression of GSDMD mRNA in myocardial tissues by RT-qPCR. In Figure 3A, GSDMD exhibited the up-regulated mRNA expression in myocardial I/R tissues than sham specimens. But silencing PVT1 markedly decreased the expression of GSDMD mRNA in myocardial I/R tissues. Immunohistochemistry was carried out to examine the expression of GSDMD protein. In comparison to sham mice, increased GSDMD expression was found in myocardial I/R tissues, which was distinctly lowered by PVT1 knockdown (Figures 3B,C). Western blot showed that I/R or PVT1 knockdown did not affect GSDMD-FL expression but affected GSDMD-N expression (Figures 3D–F). GSDMD-N expression was distinctly up-regulated in myocardial I/R tissues, which was lowered by PVT1 knockdown. Above data suggested that GSDMD was required for myocardial I/R-induced pyroptosis and silencing PVT1 alleviated GSDMD-induced pyroptosis in myocardial I/R mice.

### Suppression of Pyroptosis by lncRNA PVT1 Knockdown Involves TLR4/MyD88/NF-κB/NLRP3 Inflammasome Pathway

**Figure 4:**
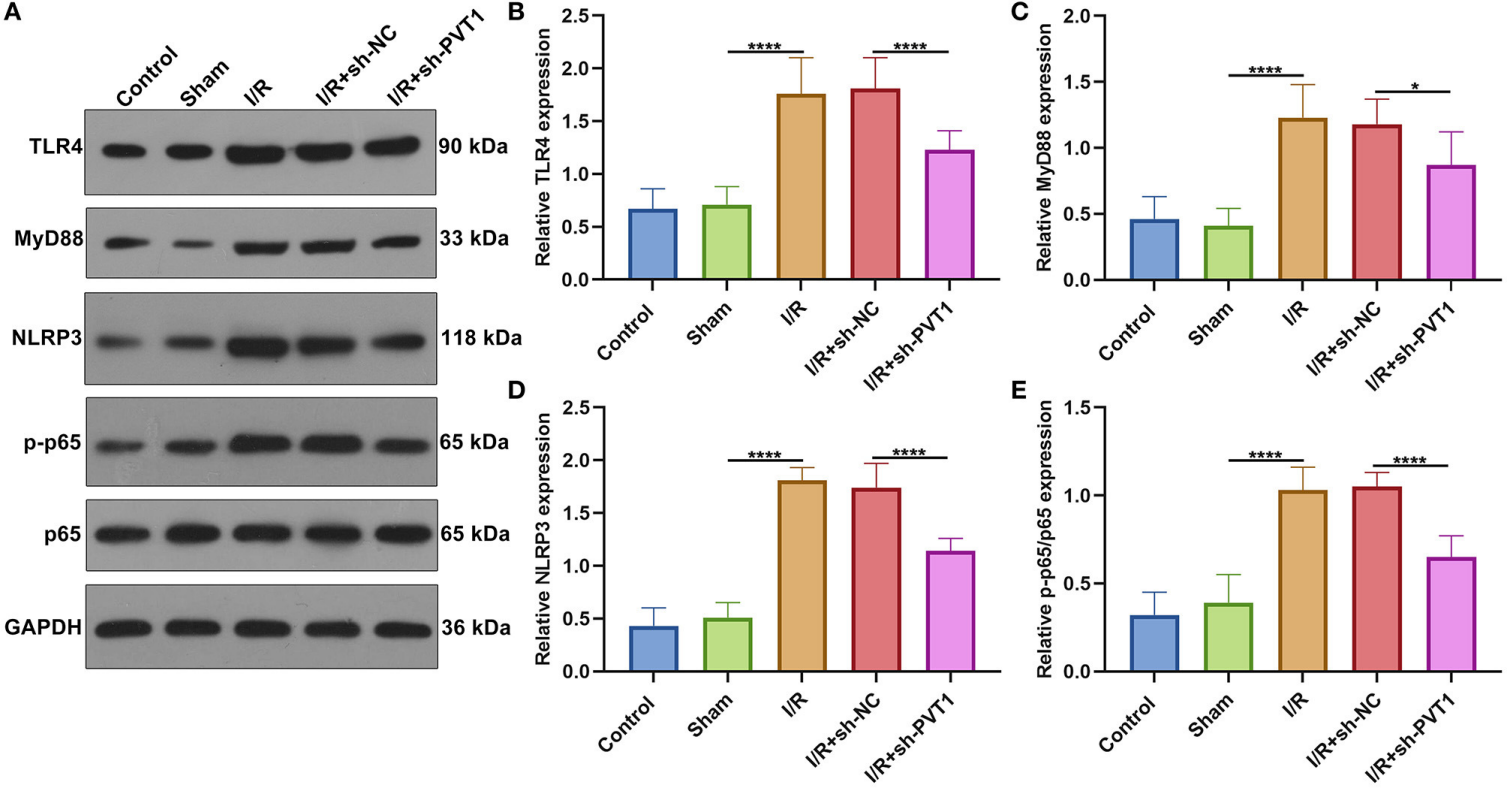
Suppression of pyroptosis by lncRNA PVT1 knockdown involves TLR4/MyD88/NF-κB/NLRP3 inflammasome pathway in myocardial I/R tissues. **(A–E)** Western blot of **(B)** TLR4, **(C)** MyD88, **(D)** NLRP3, and **(E)** p-p65/p65 expressions in myocardial tissues of control, sham, I/R, I/R + sh-NC, and I/R + sh-PVT1 groups. Mice in the I/R + sh-NC and I/R + sh-PVT1 groups were injected by 10 μL sh-NC or sh-PVT1 via Lipofectamine 2000 reagent. After 48 h, myocardial I/R model was constructed. No treatment was done for mice in the control group. The chest of mice in the sham operation group was only opened, but was threaded the corresponding part without ligating the LAD. There were 10 mice in each group. *p < 0.05; ****p < 0.0001.

Increasing evidence suggests that inhibition of the TLR4/MyD88/NF-κB/NLRP3 inflammasome pathway may reduce myocardial infarction-induced pyroptosis (9, 21). The activation of TLR4/MyD88/NF-κB/NLRP3 inflammasome pathway was examined in myocardial tissues by western blot (Figure 4A) (22). Our western blot showed that the expression of TLR4 (Figure 4B), MyD88 (Figure 4C), NLRP3 (Figure 4D), and p-p65/p65 (Figure 4E) was distinctly elevated in myocardial I/R tissues than sham specimens. However, their expression was markedly suppressed by PVT1 knockdown. These data suggested that suppression of pyroptosis through PVT1 knockdown involved TLR4/MyD88/NF-κB/NLRP3 inflammasome pathway in myocardial I/R tissues.

### LncRNA PVT1 Knockdown Ameliorates I/R-Induced Apoptosis and Caspase1 Involving Pyroptosis in Myocardial Tissues

**Figure 5:**
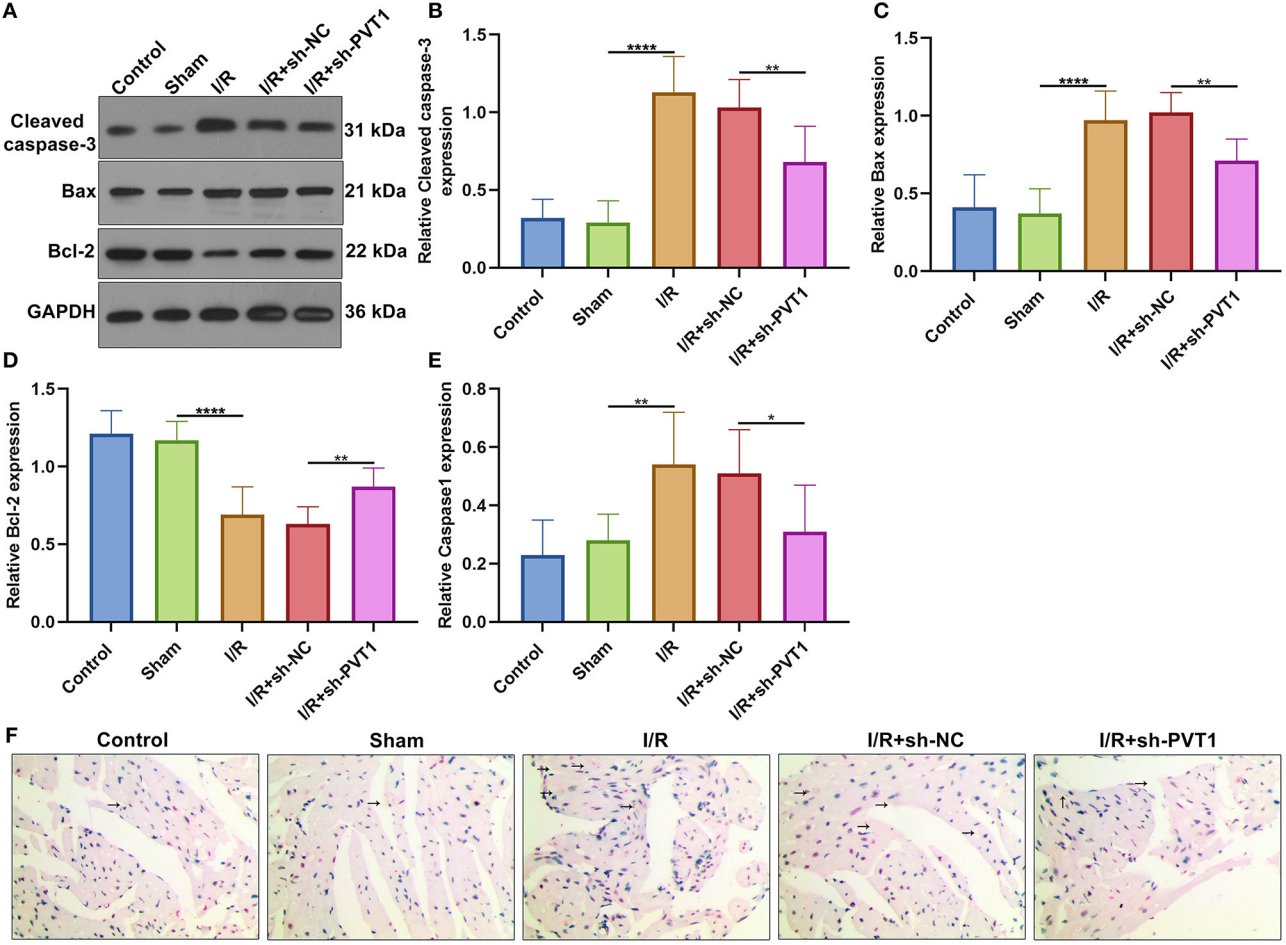
LncRNA PVT1 knockdown suppresses I/R-induced apoptosis and Caspase1 involving pyroptosis in myocardial tissues. **(A–D)** Western blot of the expression of **(B)** Cleaved caspase-3, **(C)** Bax, and **(D)** Bcl-2 proteins in myocardial tissues of five groups: control, sham, I/R, I/R + sh-NC, and I/R + sh-PVT1 groups. Mice in the I/R + sh-NC and I/R + sh-PVT1 groups were injected by 10 μL sh-NC or sh-PVT1 via Lipofectamine 2000 reagent. After 48 h, myocardial I/R model was constructed. No treatment was done for mice in the control group. The chest of mice in the sham operation group was only opened, but was threaded the corresponding part without ligating the LAD. There were 10 mice in each group. **(E,F)** Immunohistochemistry of the expression of Caspase1 protein in myocardial tissues of above groups. Bar = 100 μm. Arrow highlights the positive staining of Caspase1. *p < 0.05; **p < 0.01; ****p < 0.0001.

We also investigated whether PVT1 knockdown affected cardiomyocyte apoptosis. Western blot was presented for examining Cleaved caspase-3, Bax, and Bcl-2 expression in myocardial tissues (Figure 5A). We found that the expression of Cleaved caspase-3 (Figure 5B) and Bax proteins (Figure 5C) was distinctly increased as well as Bcl-2 expression (Figure 5D) was markedly decreased in myocardial I/R tissues than sham tissues. Moreover, silencing PVT1 markedly alleviated I/R-induced the increase in Cleaved caspase-3 and Bax proteins as well as the decrease in Bcl-2 protein in myocardial tissues. Pyrolysis is dependent on Caspase1. Here, Caspase1 expression was examined by immunohistochemistry. The up-regulation of Caspase1 was found in myocardial I/R tissues than sham tissues, which was suppressed when silencing PVT1 expression (Figures 5E,F).

### LncRNA PVT1 Knockdown Alleviates H/R-Induced Apoptosis in Cardiomyocytes

**Figure 6:**
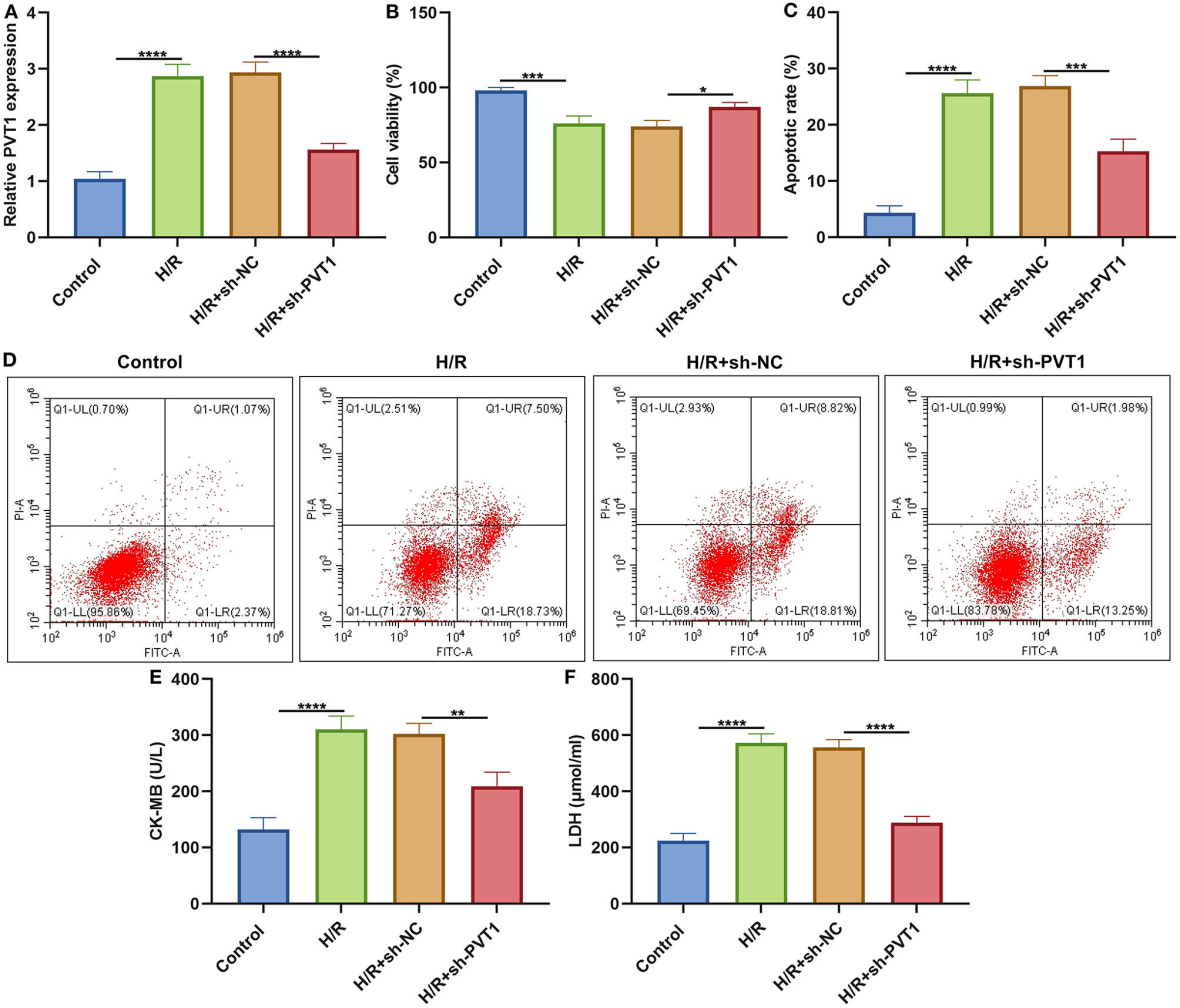
LncRNA PVT1 knockdown alleviates H/R-induced apoptosis in H9C2 cells. **(A)** RT-qPCR of PVT1 expression in H9C2 cells in four groups: control, H/R, H/R + sh-NC, and H/R + sh-PVT1 groups. H9C2 cells in the H/R, H/R + sh-NC, and H/R + sh-PVT1 groups were treated by H/R for 4 h. For the H/R + sh-NC or H/R + sh-PVT1 groups, H9C2 cells (5 × 10^4^) were transfected by 1 μg sh-NC or 1 μg sh-PVT1 for 48 h. No treatment was done for H9C2 cells in the control group. **(B)** CCK-8 for the cell viability of H9C2 cells in above groups. **(C,D)** Flow cytometry of apoptotic levels of H9C2 cells in each group. **(E,F)** ELISA of the levels of CK-MB and LDH in the supernatant of H9C2 cells in above groups. Each experiment was repeated three times. *p < 0.05; **p < 0.01; ***p < 0.001; ****p < 0.0001.

This study established H/R-induced cellular models. The up-regulation of PVT1 was detected in H/R-induced H9C2 cells than controls (Figure 6A). After transfection with sh-PVT1, PVT1 expression was markedly suppressed. CCK-8 results showed that H/R markedly lowered cellular viability of H9C2 cells (Figure 6B). But PVT1 knockdown ameliorated the decrease in cell viability induced by H/R. In Figures 6C,D, H/R treatment markedly elevated apoptotic levels of H/R-treated H9C2 cells. Nevertheless, silencing PVT1 markedly lowered the apoptotic levels of H/R-induced H9C2 cells.

### LncRNA PVT1 Knockdown Ameliorates H/R-Induced Pyroptosis in Cardiomyocytes

**Figure 7:**
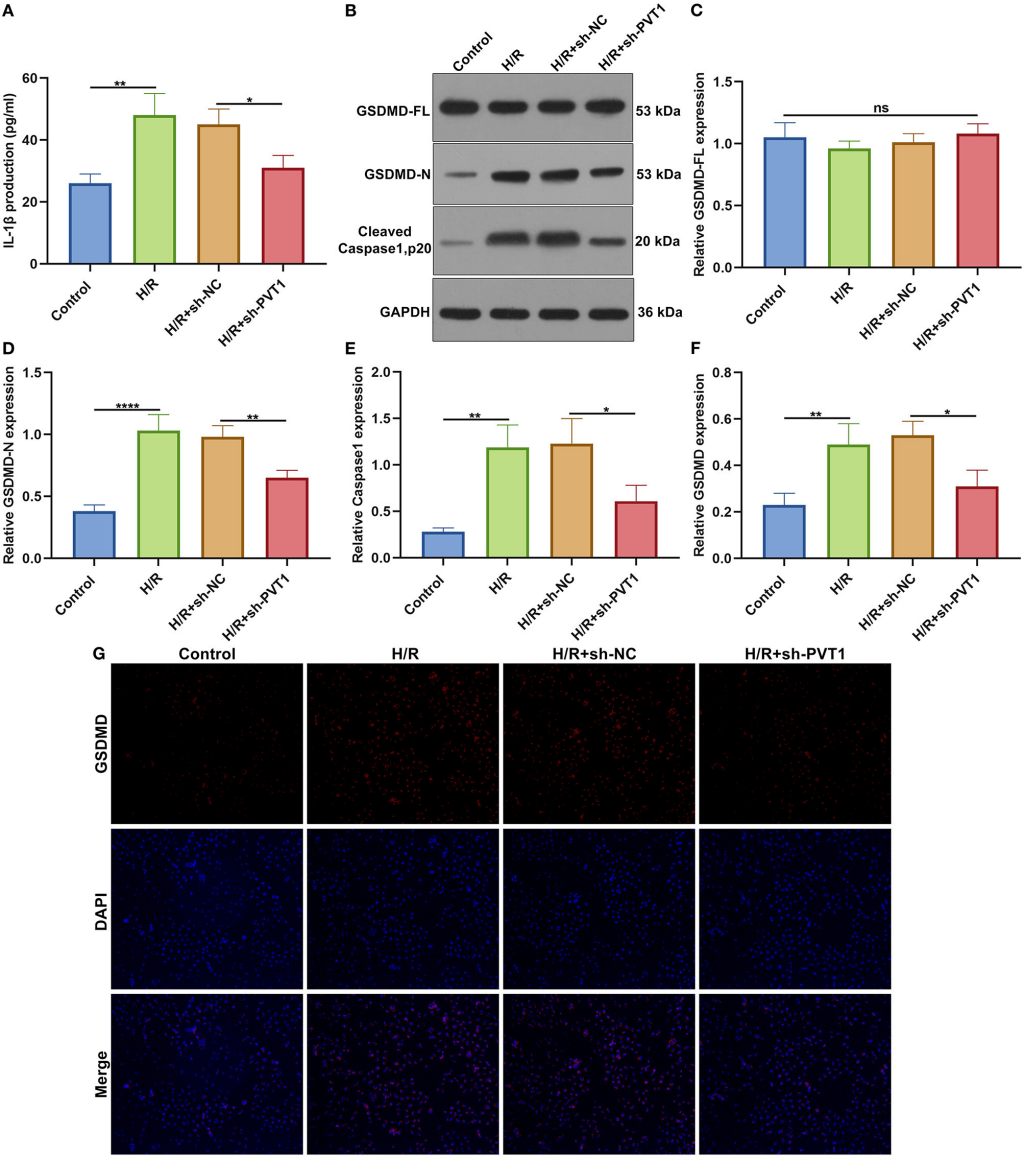
LncRNA PVT1 knockdown ameliorates H/R-induced pyroptosis in cardiomyocytes. **(A)** ELISA for the levels of IL-1β production in H9C2 cells in four groups: control, H/R, H/R + sh-NC, and H/R + sh-PVT1 groups. H9C2 cells in the H/R, H/R + sh-NC, and H/R + sh-PVT1 groups were treated by H/R for 4 h. For the H/R + sh-NC or H/R + sh-PVT1 groups, H9C2 cells (5 × 10^4^) were transfected by 1 μg sh-NC or 1 μg sh-PVT1 for 48 h. No treatment was done for H9C2 cells in the control group. **(B–E)** Western blot for **(C)** GSDMD-FL, **(D)** GSDMD-N, and **(E)** cleaved Caspase1 (p20) expressions in H9C2 cells in above groups. **(F,G)** Immunofluorescence for the expression of GSDMD in H9C2 cells in above groups. Each experiment was repeated three times. Ns: not significant; *p < 0.05; **p < 0.01; ****p < 0.0001.

ELISA was presented for testing the levels of CK-MB and LDH in the supernatant of H9C2 cells. Our data showed that H/R markedly elevated CK-MB (Figure 6E) and LDH (Figure 6F) production in the supernatant, which was alleviated by PVT1 knockdown. Furthermore, we tested IL-1β production in the cell supernatant via ELISA. IL-1β was markedly up-regulated in H/R-induced H9C2 cells than controls (Figure 7A). Meanwhile, its production was significantly inhibited under silencing PVT1 in H9C2 cells. As shown in western blot, H/R or PVT1 knockdown did not alter GSDMD-FL expression in H9C2 cells (Figures 7B,C). But H/R treatment distinctly elevated the expression of GSDMD-N, which was markedly alleviated by PVT1 knockdown (Figure 7D). Moreover, Caspase1 expression was significantly up-regulated in H/R-induced H9C2 cells than controls (Figure 7E). Nevertheless, sh-PVT1 transfection significantly lowered its level H/R-treated cardiomyocytes. Immunofluorescence assays were also carried out to verify GSDMD expression in H9C2 cells. Consistently, the up-regulation of GSDMD was mediated by H/R treatment, which was lowered through PVT1 knockdown in H9C2 cells (Figures 7F,G).

### Overexpression of lncRNA PVT1 Promotes H/R-Mediated Pyroptosis in Cardiomyocytes

**Figure 8:**
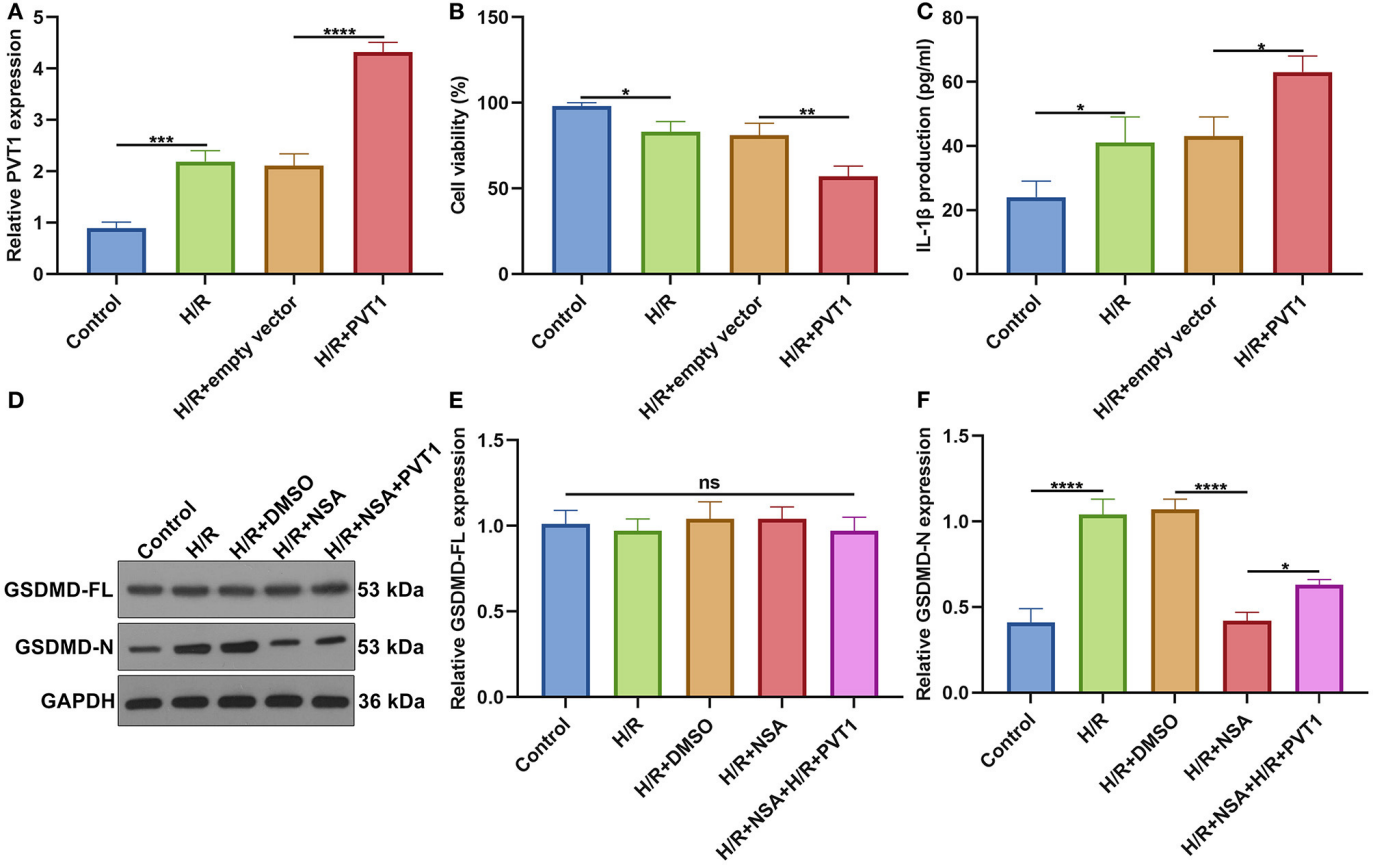
Overexpression of lncRNA PVT1 promotes H/R-mediated pyroptosis in H9C2 cells. **(A)** RT-qPCR for the expression of PVT1 in H9C2 cells in four groups: control, H/R, H/R + empty vector, and H/R + PVT1 groups. H9C2 cells in the H/R, H/R + empty vector, and H/R + PVT1 groups were treated by H/R for 4 h. For the H/R + empty vector and H/R + PVT1 groups, H9C2 cells were transfected by plasmids of 500 ng pcDNA3.1 vector (empty vector) or 500 ng PVT1 for 48 h. No treatment was done for H9C2 cells in the control group. **(B)** CCK-8 for the cell viability of H9C2 cells in above groups. **(C)** ELISA for IL-1β levels in the cell supernatant in above groups. **(D–F)** Western blot of **(E)** GSDMD-FL and **(F)** GSDMD-N expressions in H9C2 cells in five groups: control, H/R group, H/R + DMSO, H/R + NSA, and H/R + NSA + PVT1 groups. H9C2 cells were treated with 25 nM NSA, followed by plasmids of 500 ng PVT1 and H/R treatment. Each experiment was repeated three times. Ns: not significant; *p < 0.05; **p < 0.01; ***p < 0.001; ****p < 0.0001.

For further investigating the influence of PVT1 on H/R-induced pyroptosis, H9C2 cells were transfected by PVT1 overexpression vector. In Figure 8A, RT-qPCR demonstrated that H/R-induced PVT1 up-regulation was enhanced by PVT1 overexpression vector transfection in H9C2 cells. CCK-8 showed that PVT1 overexpression promoted the decrease in cell viability induced by H/R (Figure 8B). Furthermore, its overexpression strengthened the increase in IL-1β production induced by H/R treatment in H9C2 cells (Figure 8C). Our western blot showed that pyroptosis inhibitor NSA did not affect GSDMD-FL expression but significantly suppressed the expression of GSDMD-N in H/R-induced H9C2 cells (Figures 8D–F). Nevertheless, PVT1 overexpression significantly alleviated the inhibitory effect of NSA on GSDMD-N expression in H9C2 cells.

## Discussion

In our study, lncRNA PVT1 expression displayed distinct up-regulation in I/R-induced myocardial tissues as well as H/R-induced H9C2 cells. Consistently, Mao et al. reported the up-regulation of PVT1 in H/R-induced AC16 cells (23). In previous studies, lncRNA MALAT1 knockdown decreased cTnI, LDH and CK-MB levels for oxygen-glucose deprivation and reoxygenation (OGD/R)-mediated cardiomyocytes (24). Silencing HIF1A-AS1 could lower serum BNP, cTnI, LDH and CK-MB levels in myocardial I/R rat models (25). Furthermore, targeting FOXD3-AS1 markedly decreased the levels of cTnI and CK-MB in OGD/R-induced H9C2 cells (26). Here, in I/R-induced mouse models, we found that PVT1 knockdown significantly alleviated the expression of myocardial damage markers including cTnI, LDH, BNP as well as CK-MB in serum samples from I/R-induced mouse models. H&E and Masson staining confirmed that silencing PVT1 could alleviate I/R-induced myocardial damage.

The α-MHC and β-MHC are major contractile proteins of cardiomyocytes (27). The β-MHC is mainly expressed in the embryonic stage of mice and is almost replaced by α-MHC after birth (28). However, the expression will increase under pathological conditions such as heart failure and increased angiotensin II. Therefore, conversion of α-MHC to β- MHC is also regarded as an important sign of heart failure (29). PVT1 knockdown significantly increased α-MHC and decreased β-MHC expressions in I/R-induced myocardial tissues, indicating that targeting PVT1 could improve cardiac function of mice following I/R damage. As previously reported, silencing PVT1 lowered β-MHC expression in cardiac hypertrophic mouse models (30). PVT1 knockdown markedly reduced the production and secretion of pro-inflammatory cytokines TNF-α, IL-1β and IL-6 in I/R-induced myocardial tissue specimens. TNF-α antagonism may alleviate myocardial I/R damage through elevating adiponectin expression (31). Our data indicated the anti-inflammatory effect of PVT1 knockdown on myocardial I/R damage. GSDMD-induced pyroptosis of cardiomyocytes accelerates myocardial I/R damage (10). A recent study demonstrated that PVT1 may modulate NLRP3-induced pyroptosis in septic acute kidney damage (32). Here, we found that silencing PVT1 alleviated GSDMD-N not GSDMD-FL expression in I/R-induced myocardial tissue specimens as well as H/R-treated cardiomyocytes. Thus, PVT1 knockdown could ameliorate GSDMD-mediated pyroptosis in cardiomyocytes.

It has been confirmed that TLR4/MyD88/NF-κB/NLRP3 inflammasome pathway is responsible for pyroptosis (9, 21). For instance, Nicorandil suppresses TLR4/MyD88/NF-κB/NLRP3 axis to alleviate pyroptosis in rats with myocardial infarction (21). Emodin reduces myocardial I/R injury-induced pyroptosis through inhibiting the TLR4/MyD88/NF-κB/NLRP3 inflammasome pathway (9). Furthermore, octreotide and melatonin reduce inflammasome-induced pyroptosis through suppressing TLR4-NF-κB-NLRP3 pathway in hepatic I/R damage (33). In this study, TLR4/MyD88/NF-κB/NLRP3 inflammasome pathway was activated in myocardial I/R tissues, consistent with a previous study (34). Silencing PVT1 decreased the activation of TLR4/MyD88/NF-κB/NLRP3 pathway. This indicated that suppression of pyroptosis through PVT1 knockdown involved TLR4/MyD88/NF-κB/NLRP3 inflammasome pathway in myocardial I/R. Except for GSDMD-induced pyroptosis, our data showed that PVT1 knockdown alleviated I/R-induced the increase in cleaved caspase3 and Bax and the decrease in Bcl-2 in myocardial tissues. For H/R-treated cardiomyocytes, silencing PVT1 elevated cellular viability as well as suppressed apoptosis. As reported, PVT1 could protect human AC16 cardiomyocytes from H/R-induced apoptosis (23). Thus, targeting PVT1 could ameliorate myocardial I/R damage via suppressing cardiomyocyte apoptosis.

Our findings proposed for the first time that inhibition of PVT1 could alleviate myocardial I/R damage through reducing GSDMD-mediated pyroptosis *in vivo* and *in vitro*, which provided a novel direction for prevention and treatment of myocardial I/R damage.

## Conclusion

Collectively, our study established myocardial I/R mouse models and H/R-induced cellular models and confirmed the up-regulation of lncRNA PVT1 following myocardial I/R damage. Silencing PVT1 ameliorated myocardial I/R damage through inhibiting GSDMD-mediated pyroptosis. Hence, PVT1 knockdown might be an alternative therapeutic strategy against myocardial I/R damage.

## Data Availability Statement

The original contributions presented in the study are included in the article/Supplementary Material, further inquiries can be directed to the corresponding authors.

## Ethics Statement

The study was approved by the Ethics Committee of Guangdong Provincial People's Hospital (GDREC2016255H).

## Author Contributions

YL, YW, and HD conceived and designed the study. CL, HS, and CC conducted most of the experiments and data analysis and wrote the manuscript. SC, QZ, DL, and JL participated in collecting data and helped to draft the manuscript. All authors reviewed and approved the manuscript.

## Funding

This work was funded by The Key Project of Natural Science Foundation of Guangdong Province (2017B030311010).

## Conflict of Interest

The authors declare that the research was conducted in the absence of any commercial or financial relationships that could be construed as a potential conflict of interest.

## Publisher's Note

All claims expressed in this article are solely those of the authors and do not necessarily represent those of their affiliated organizations, or those of the publisher, the editors and the reviewers. Any product that may be evaluated in this article, or claim that may be made by its manufacturer, is not guaranteed or endorsed by the publisher.

## Abbreviations

I/R: ischemia reperfusion
GSDMD: gasdermin D
GSDME: gasdermin E
IL-1β: interleukin 1β
IL-6: interleukin 6
TNF-α: tumor necrosis factor alpha
lncRNA: long noncoding RNA
NLRP3: NOD-like receptor protein 3
PVT1: plasmacytoma variant translocation 1
H/R: hypoxia/reoxygenation
shRNA: short hairpin RNA
NC: negative control
LAD: left anterior descending
RT-qPCR: Real-time quantitative reverse transcription PCR
ELISA: Enzyme-linked immunosorbent assay
LDH: lactate dehydrogenase
CK-MB: creative kinase isoenzyme MB
α-MHC: α-myosin heavy chain
β-MHC: β-myosin heavy chain
CCK-8: cell counting kit-8.
