# Supplementary figures and images for: LncRNA PVT1 Knockdown Ameliorates Myocardial Ischemia Reperfusion Damage via Suppressing Gasdermin D-Mediated Pyroptosis in Cardiomyocytes
Source: Front Cardiovasc Med. 2021 Sep 14;8:747802. doi: 10.3389/fcvm.2021.747802 (PMC8476808; doi:10.3389/fcvm.2021.747802)

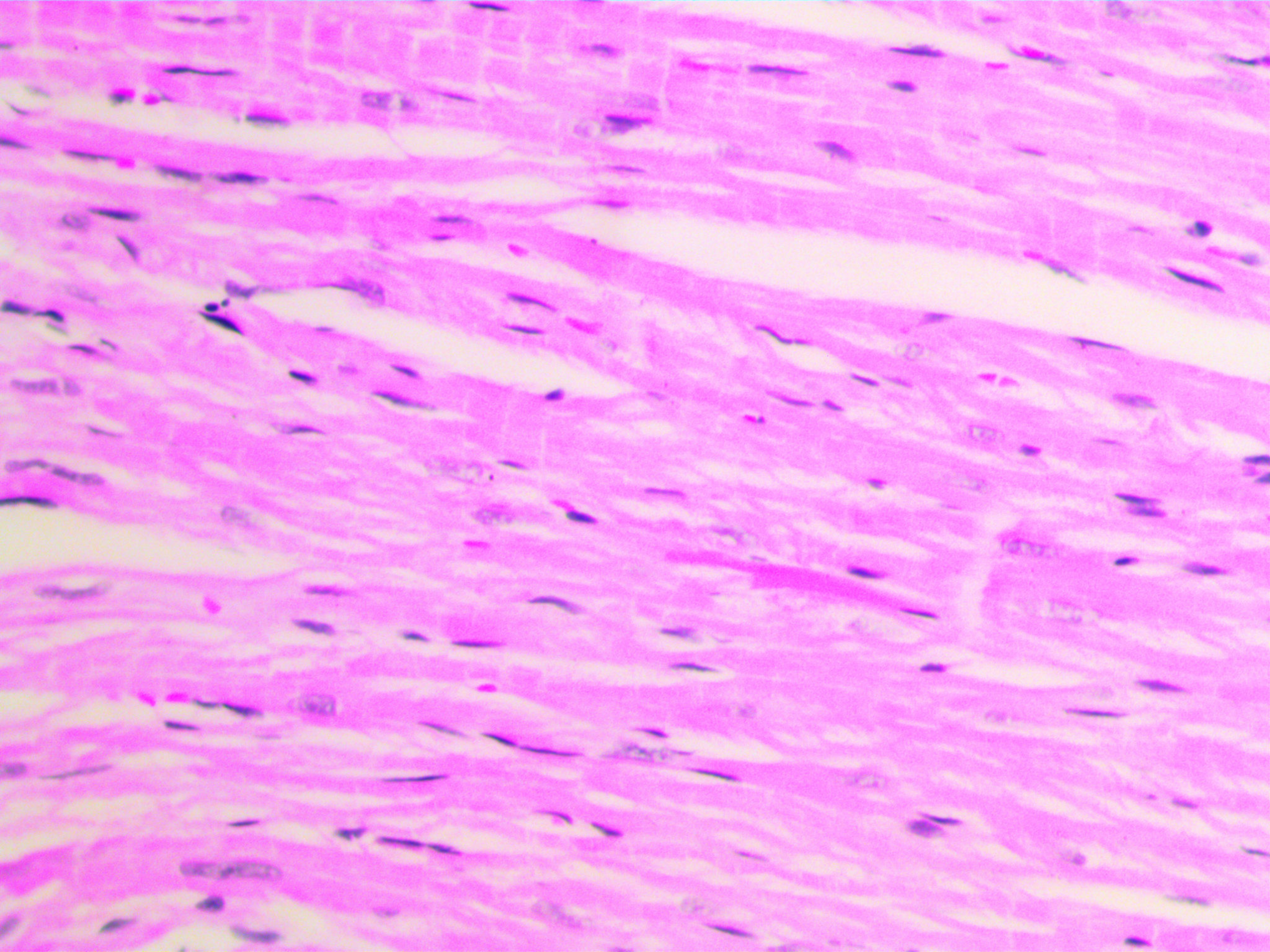

Supplement: Supplementary file 1 [file Data_Sheet_1.ZIP › Original Source Data/Figure 1/Figure 1F/Control.pdf]

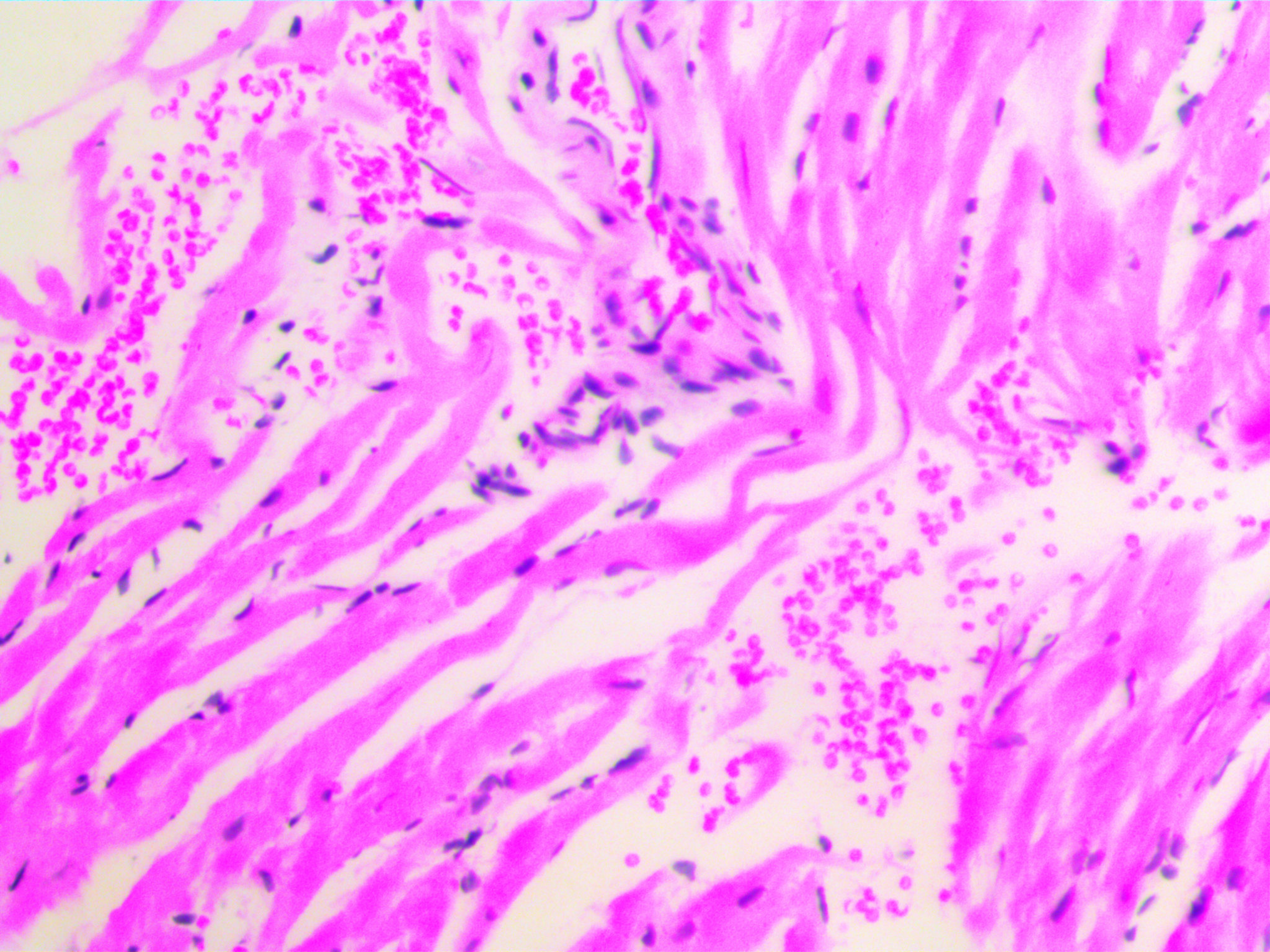

Supplement: Supplementary file 1 [file Data_Sheet_1.ZIP › Original Source Data/Figure 1/Figure 1F/IR + sh-NC.pdf]

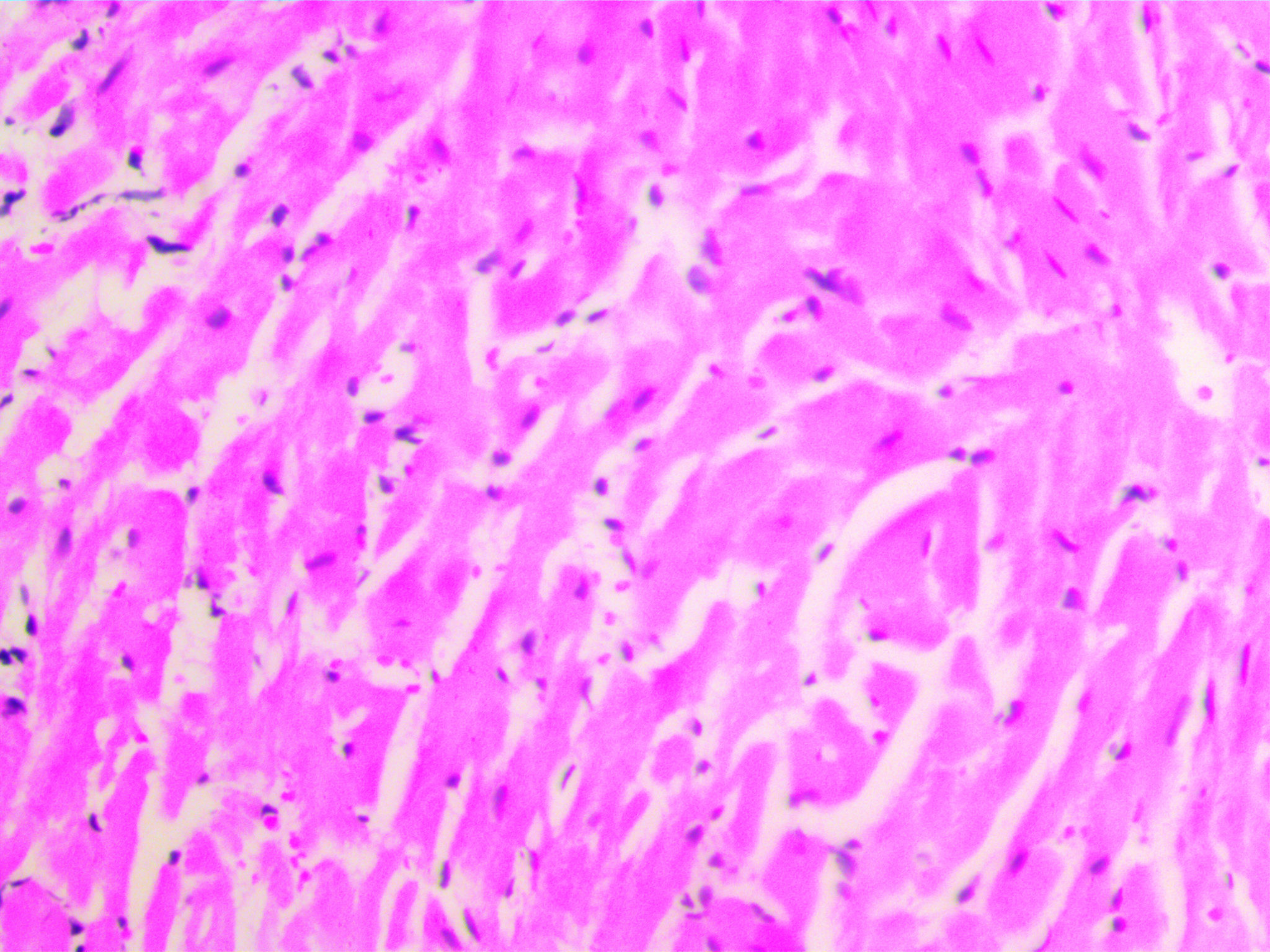

Supplement: Supplementary file 1 [file Data_Sheet_1.ZIP › Original Source Data/Figure 1/Figure 1F/IR + sh-PVT1.pdf]

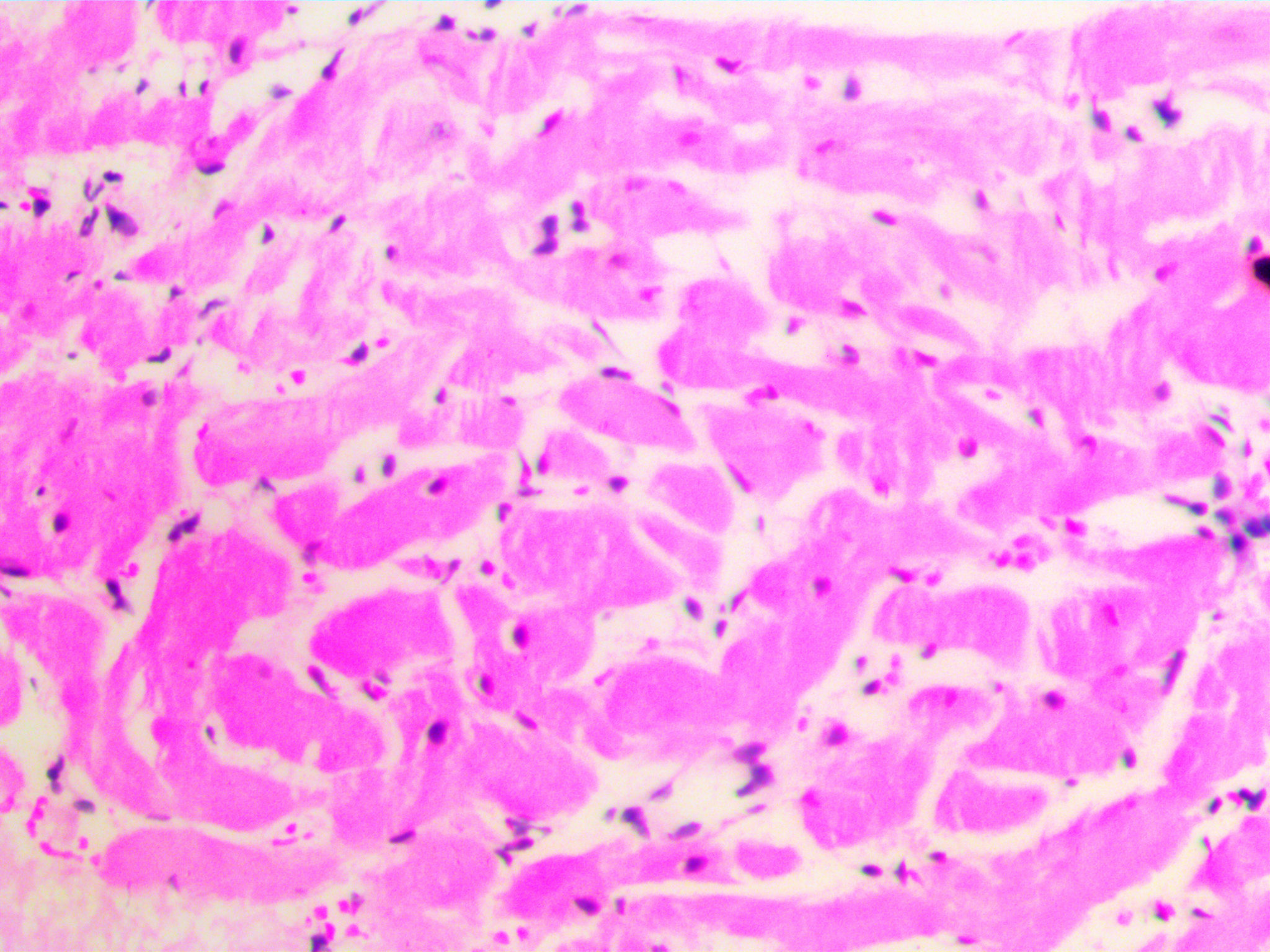

Supplement: Supplementary file 1 [file Data_Sheet_1.ZIP › Original Source Data/Figure 1/Figure 1F/IR.pdf]

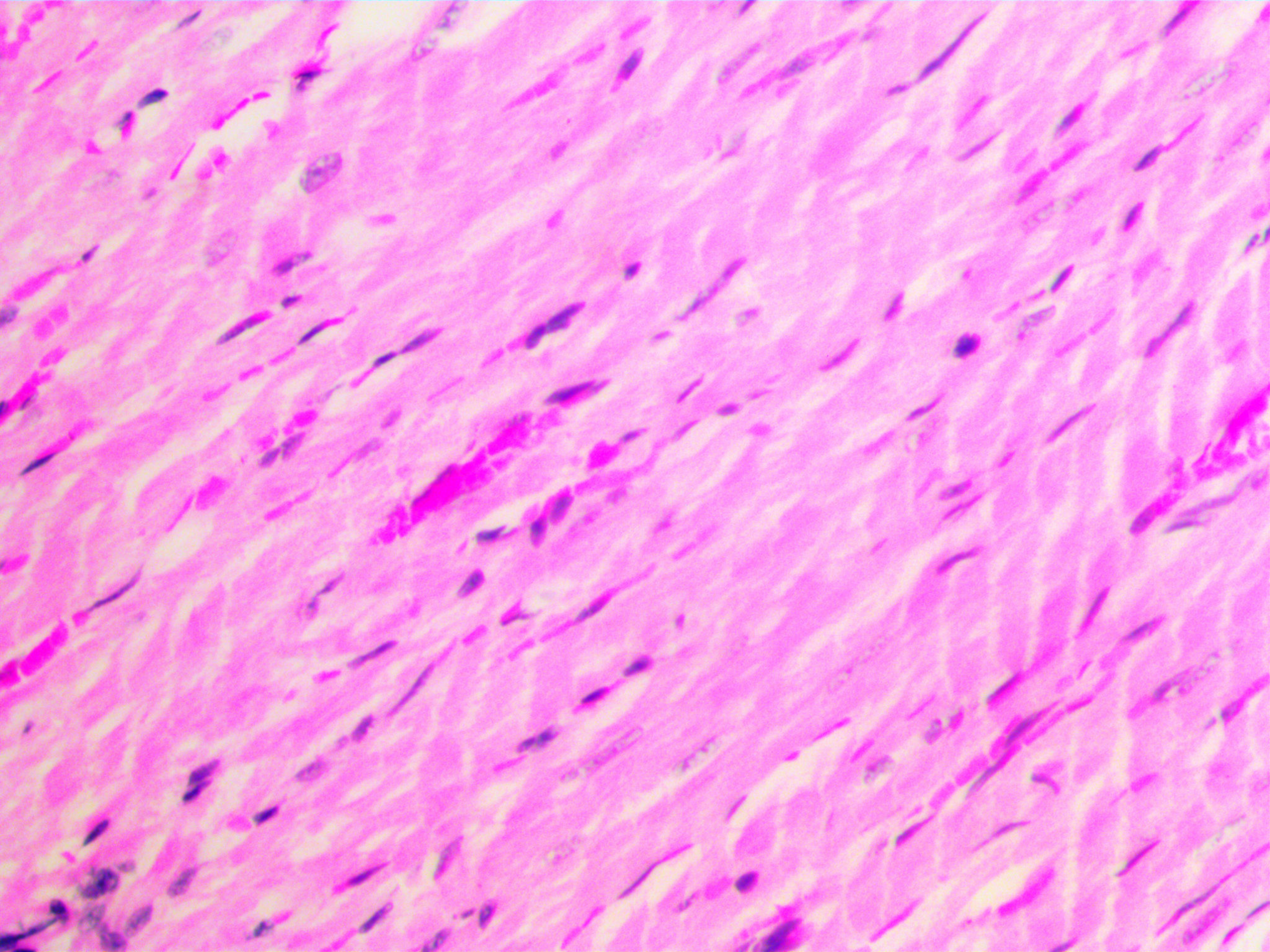

Supplement: Supplementary file 1 [file Data_Sheet_1.ZIP › Original Source Data/Figure 1/Figure 1F/Sham.pdf]

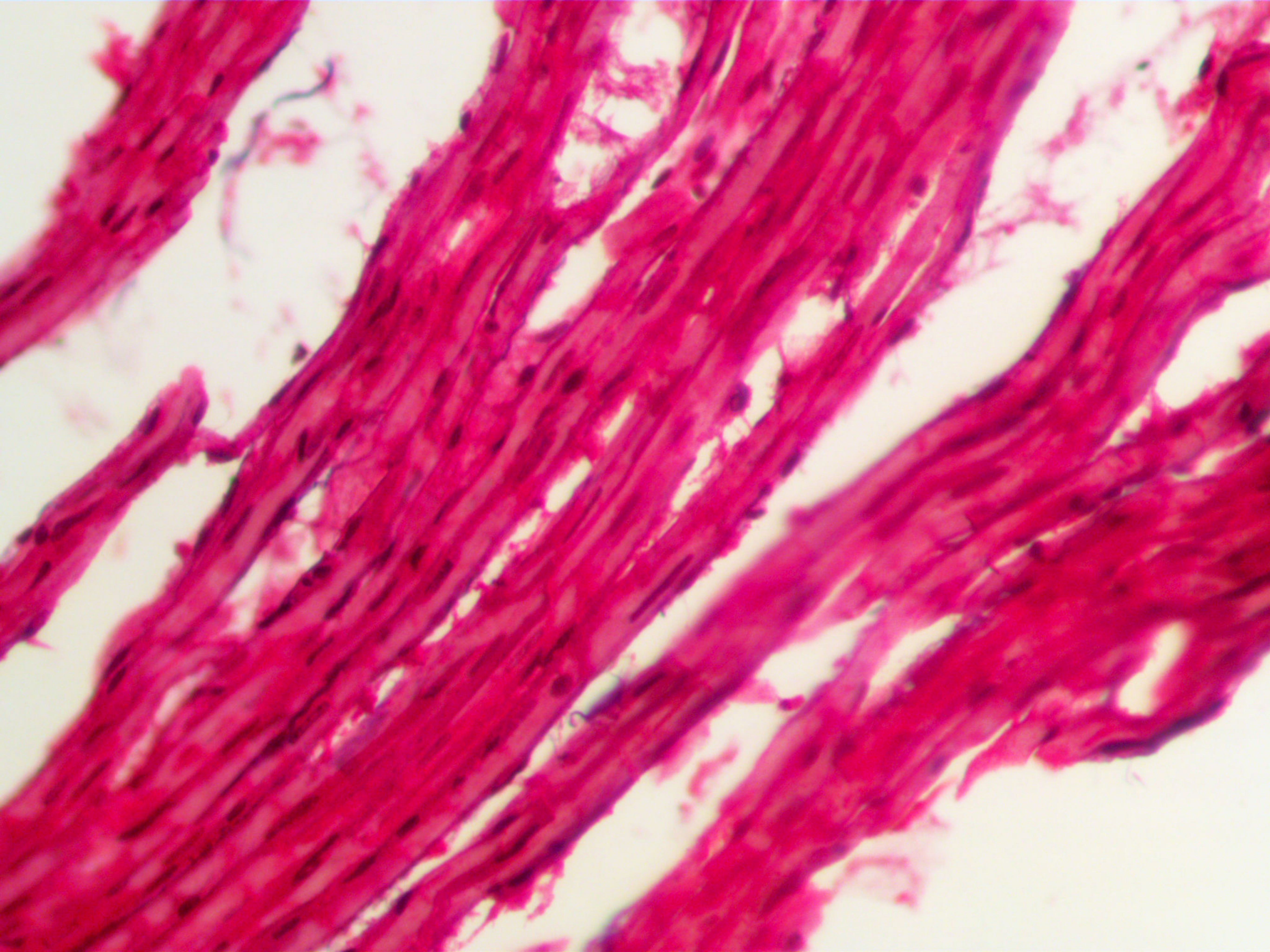

Supplement: Supplementary file 1 [file Data_Sheet_1.ZIP › Original Source Data/Figure 1/Figure 1G/Control.pdf]

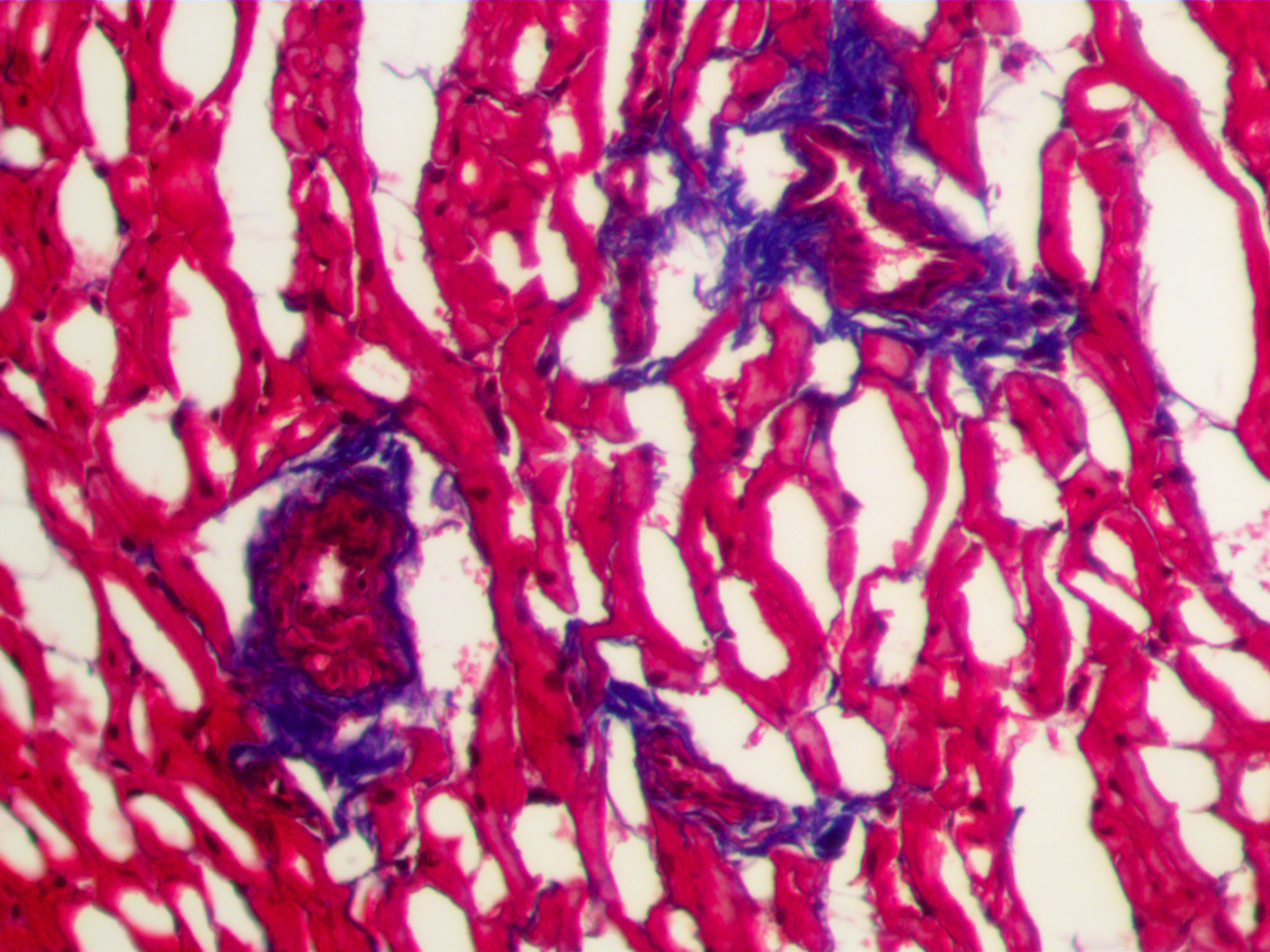

Supplement: Supplementary file 1 [file Data_Sheet_1.ZIP › Original Source Data/Figure 1/Figure 1G/IR + sh-NC.pdf]

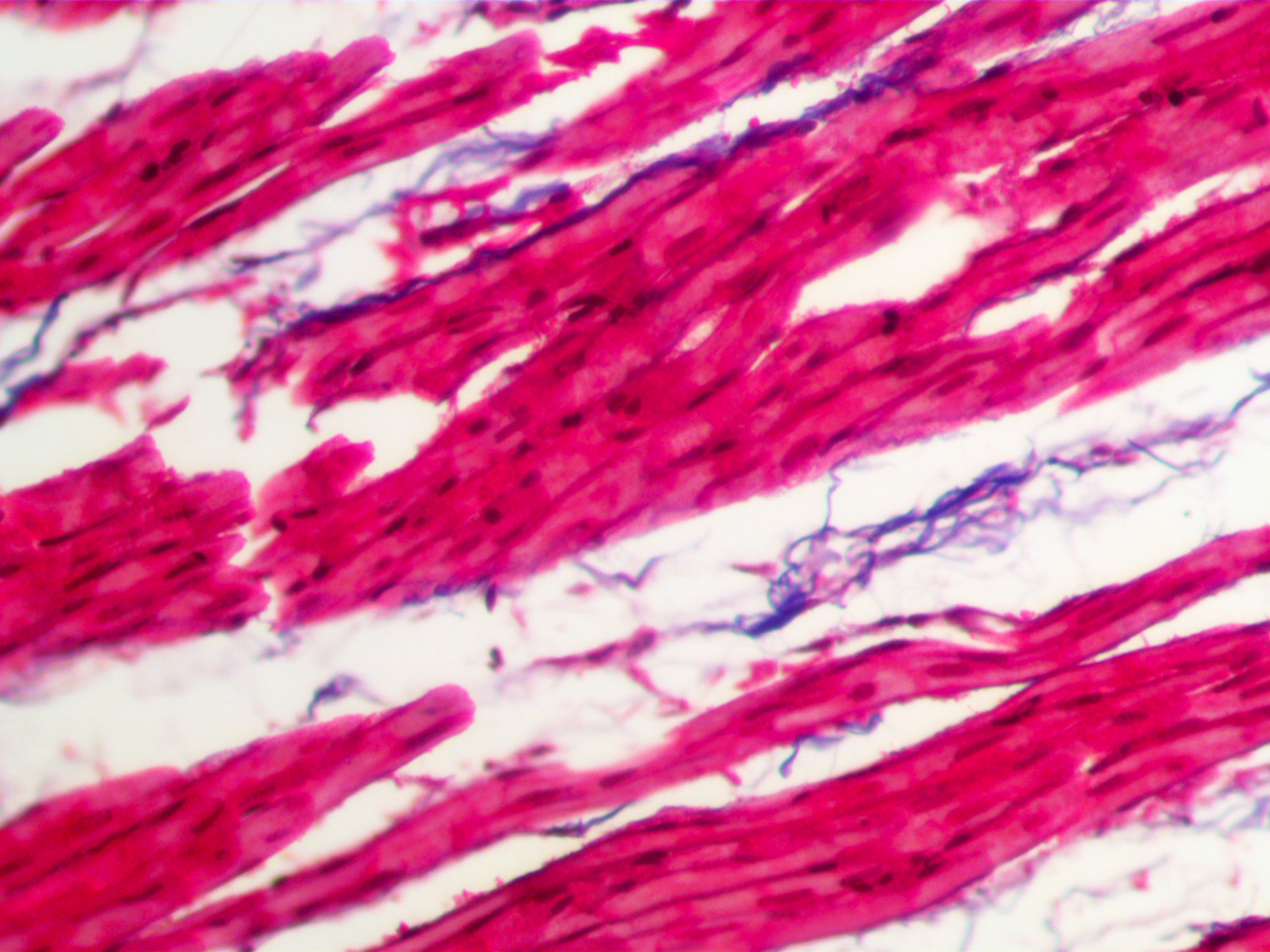

Supplement: Supplementary file 1 [file Data_Sheet_1.ZIP › Original Source Data/Figure 1/Figure 1G/IR + sh-PVT1.pdf]

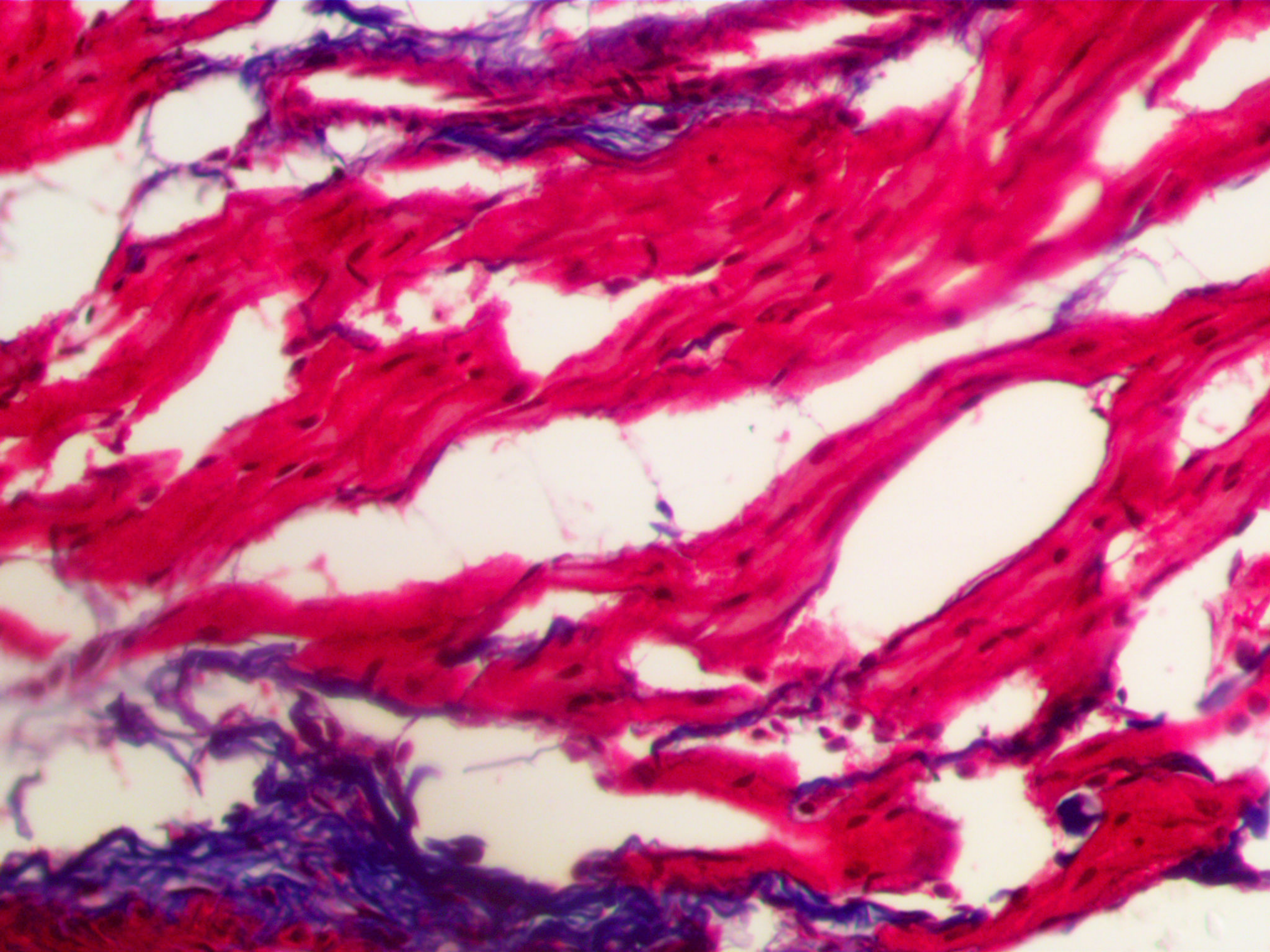

Supplement: Supplementary file 1 [file Data_Sheet_1.ZIP › Original Source Data/Figure 1/Figure 1G/IR.pdf]

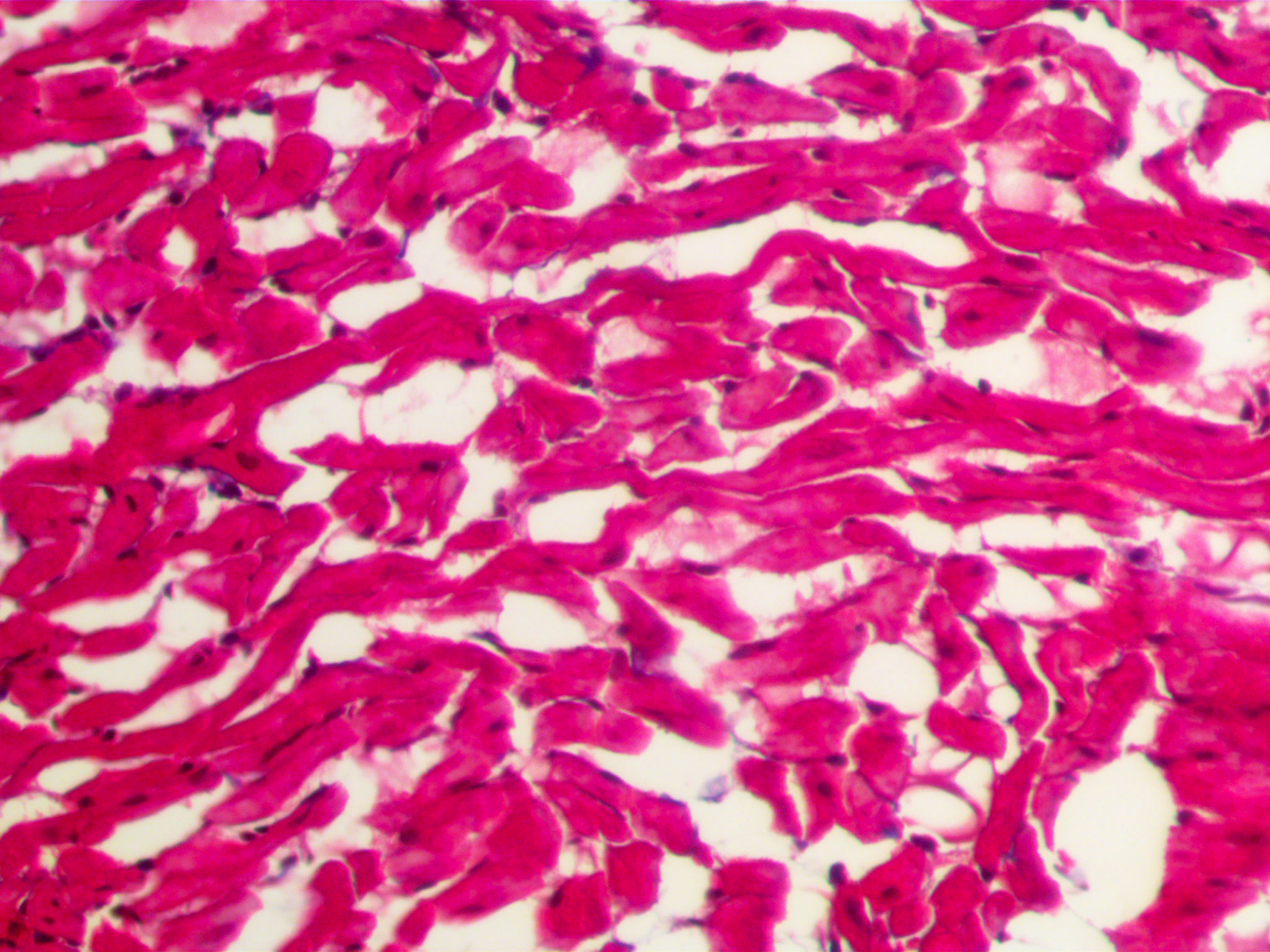

Supplement: Supplementary file 1 [file Data_Sheet_1.ZIP › Original Source Data/Figure 1/Figure 1G/Sham.pdf]

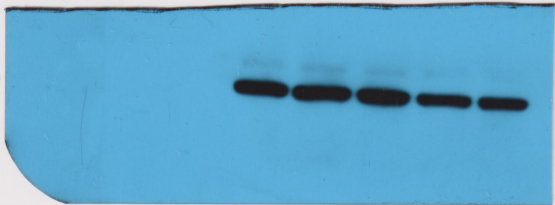

Supplement: Supplementary file 1 [file Data_Sheet_1.ZIP › Original Source Data/Figure 2/Figure 2A/GAPDH.pdf]

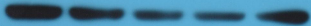

Supplement: Supplementary file 1 [file Data_Sheet_1.ZIP › Original Source Data/Figure 2/Figure 2A/α-MHC.pdf]

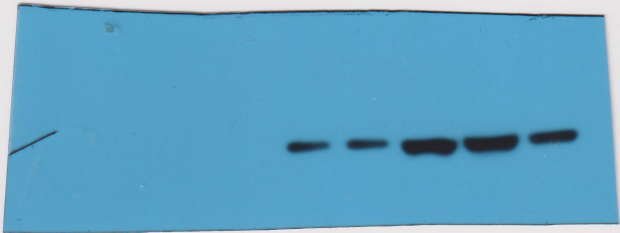

Supplement: Supplementary file 1 [file Data_Sheet_1.ZIP › Original Source Data/Figure 2/Figure 2A/β-MHC.pdf]

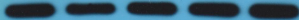

Supplement: Supplementary file 1 [file Data_Sheet_1.ZIP › Original Source Data/Figure 2/Figure 2G/GAPDH.pdf]

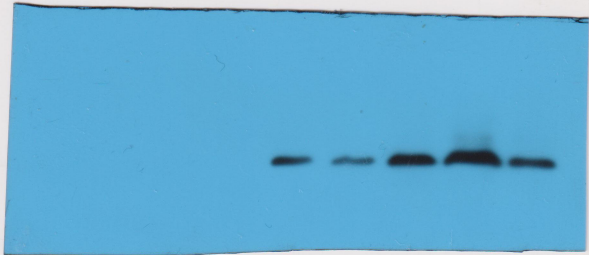

Supplement: Supplementary file 1 [file Data_Sheet_1.ZIP › Original Source Data/Figure 2/Figure 2G/IL-1β.pdf]

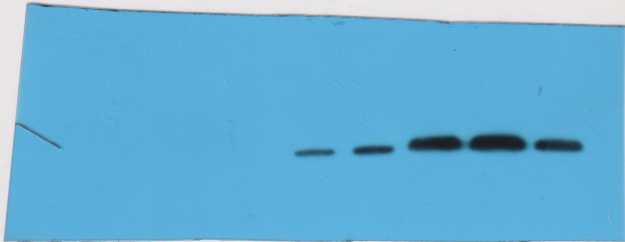

Supplement: Supplementary file 1 [file Data_Sheet_1.ZIP › Original Source Data/Figure 2/Figure 2G/IL-6.pdf]

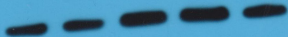

Supplement: Supplementary file 1 [file Data_Sheet_1.ZIP › Original Source Data/Figure 2/Figure 2G/TNF-α.pdf]

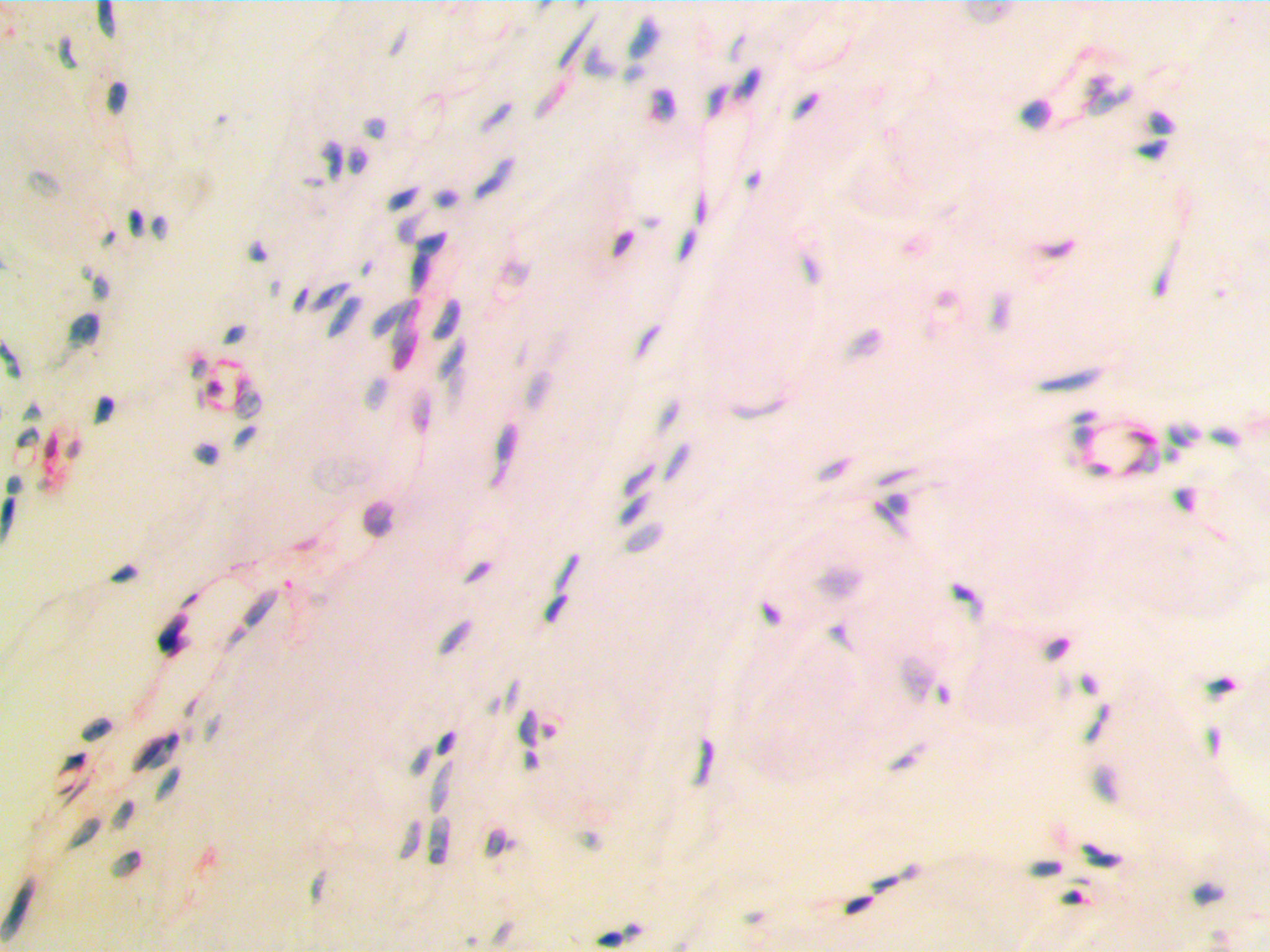

Supplement: Supplementary file 1 [file Data_Sheet_1.ZIP › Original Source Data/Figure 3/Figure 3C/Control.pdf]

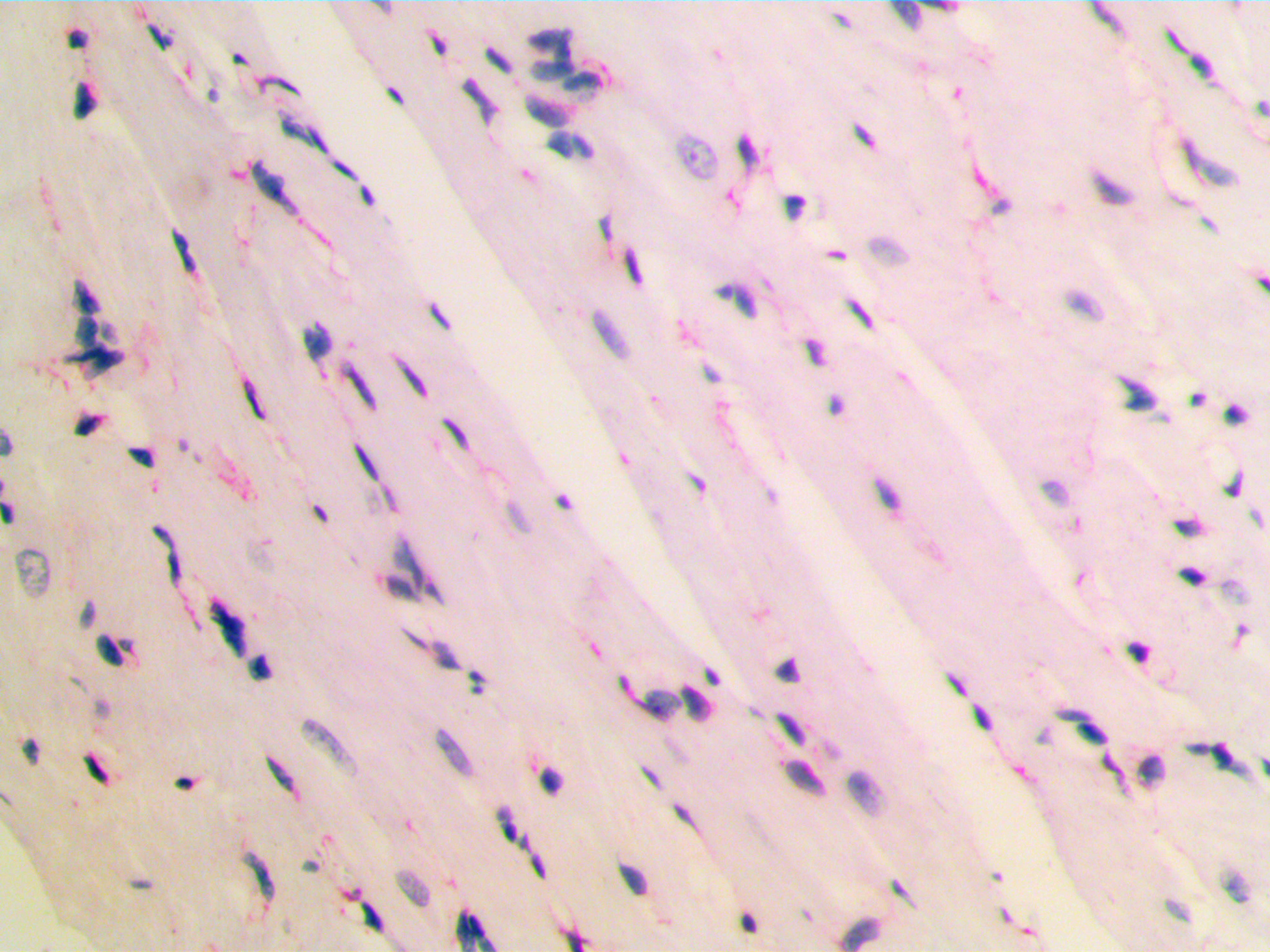

Supplement: Supplementary file 1 [file Data_Sheet_1.ZIP › Original Source Data/Figure 3/Figure 3C/IR + sh-NC.pdf]

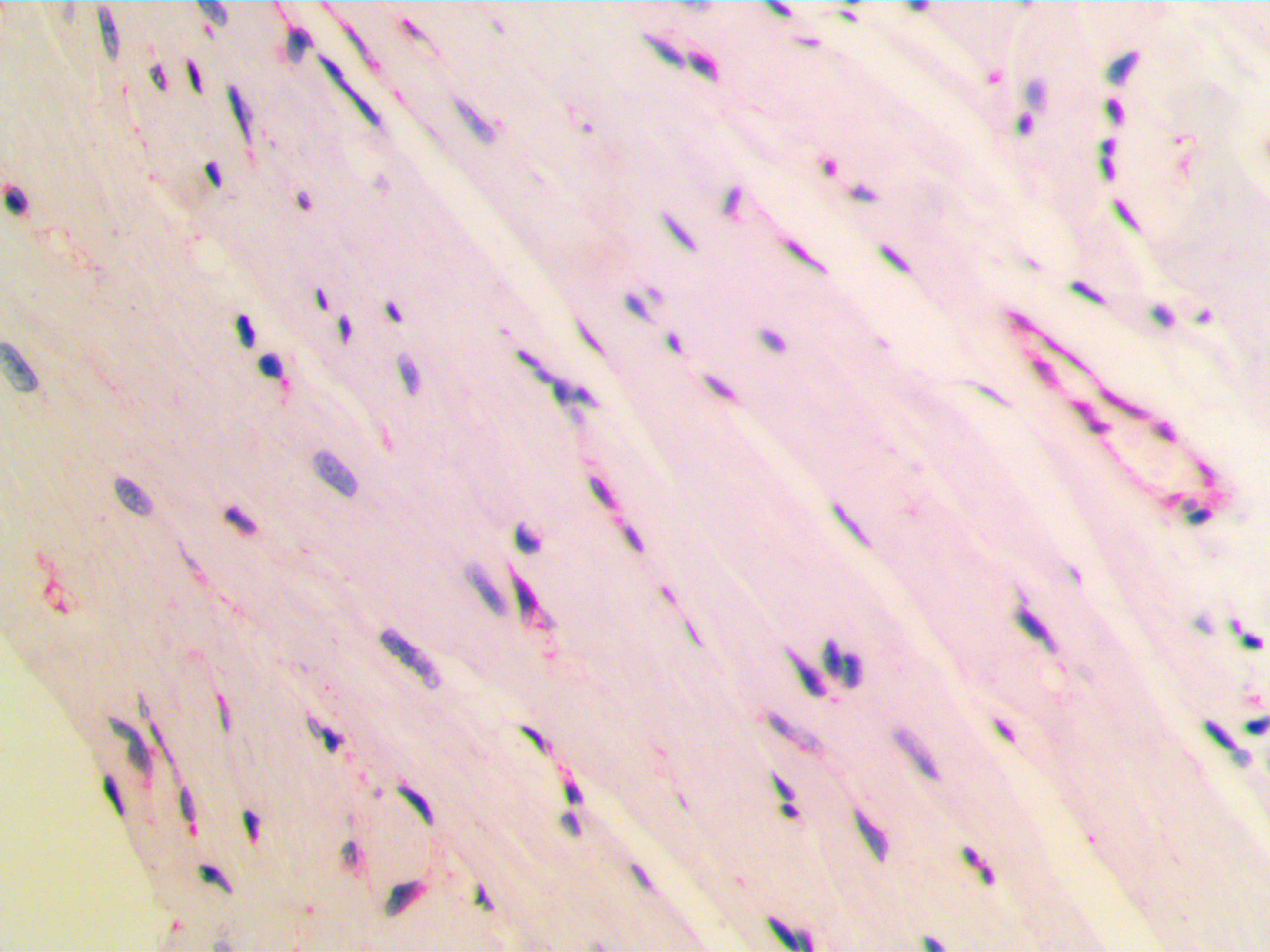

Supplement: Supplementary file 1 [file Data_Sheet_1.ZIP › Original Source Data/Figure 3/Figure 3C/IR + sh-PVT1.pdf]

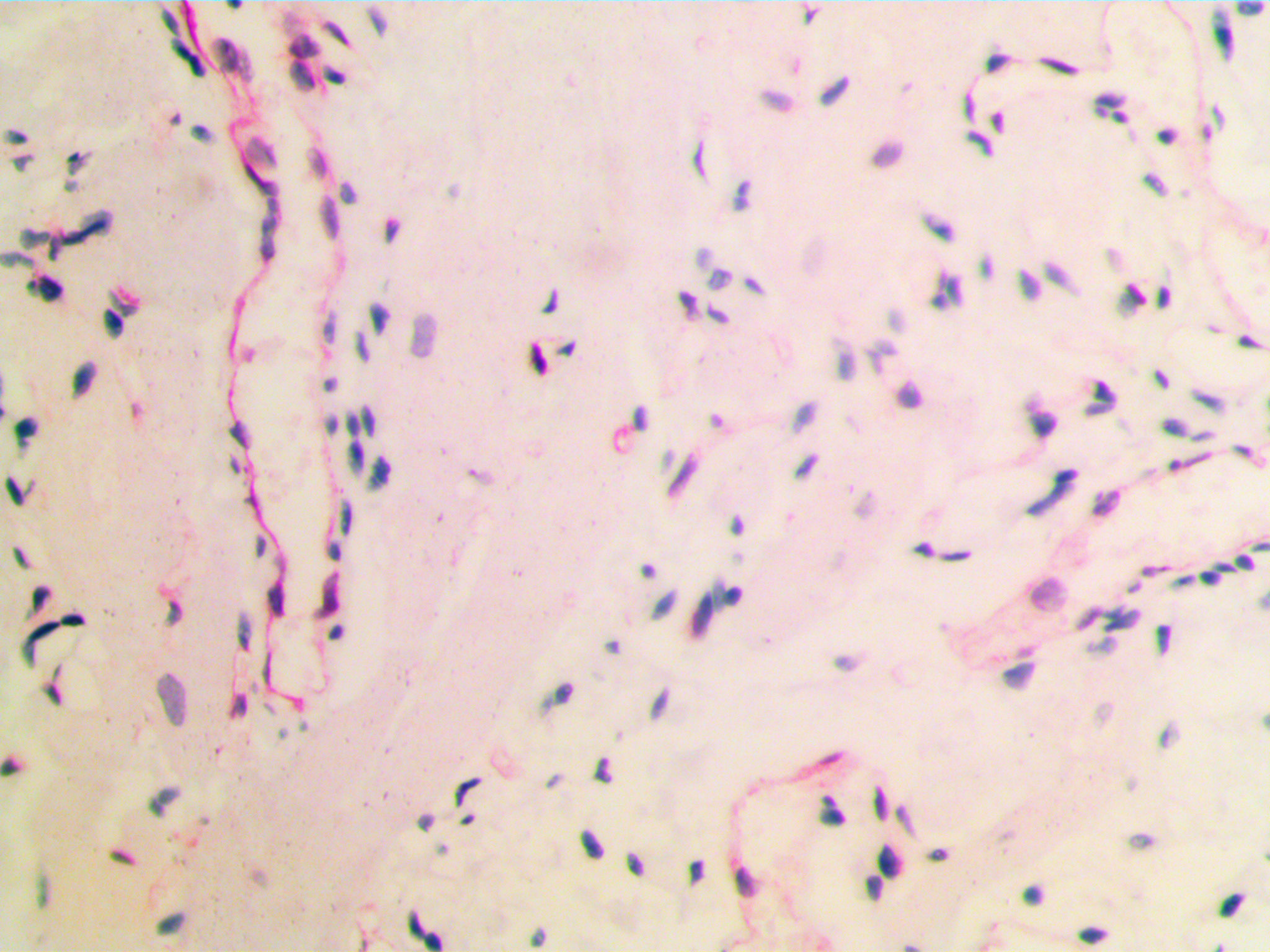

Supplement: Supplementary file 1 [file Data_Sheet_1.ZIP › Original Source Data/Figure 3/Figure 3C/IR.pdf]

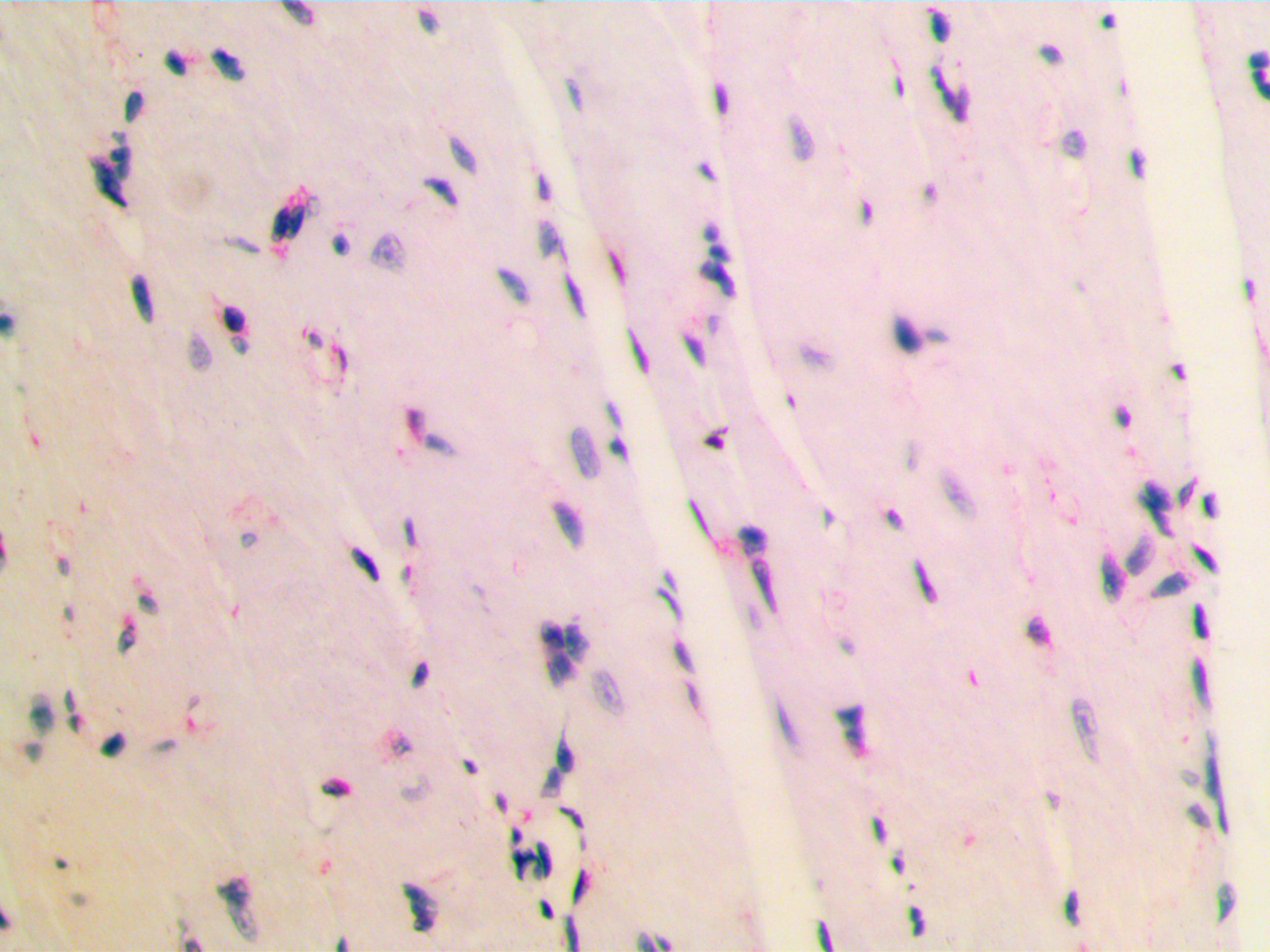

Supplement: Supplementary file 1 [file Data_Sheet_1.ZIP › Original Source Data/Figure 3/Figure 3C/Sham.pdf]

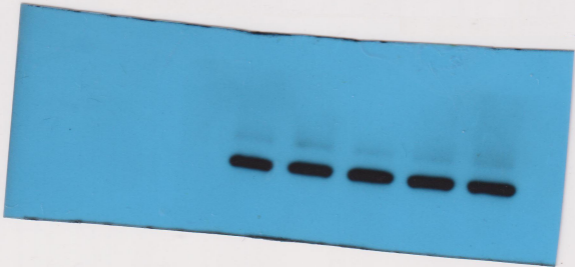

Supplement: Supplementary file 1 [file Data_Sheet_1.ZIP › Original Source Data/Figure 3/Figure 3D/GAPDH.pdf]

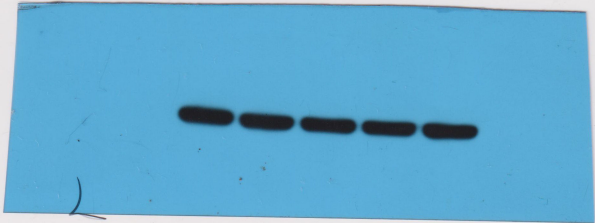

Supplement: Supplementary file 1 [file Data_Sheet_1.ZIP › Original Source Data/Figure 3/Figure 3D/GSDMD-FL.pdf]

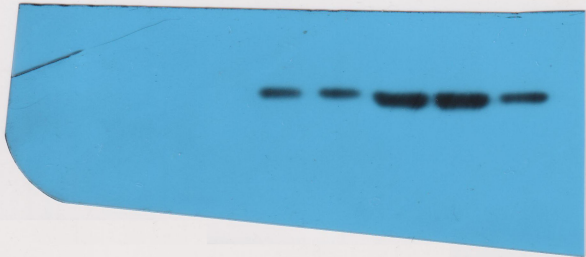

Supplement: Supplementary file 1 [file Data_Sheet_1.ZIP › Original Source Data/Figure 3/Figure 3D/GSDMD-N.pdf]

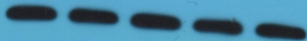

Supplement: Supplementary file 1 [file Data_Sheet_1.ZIP › Original Source Data/Figure 4/Figure 4A/GAPDH.pdf]

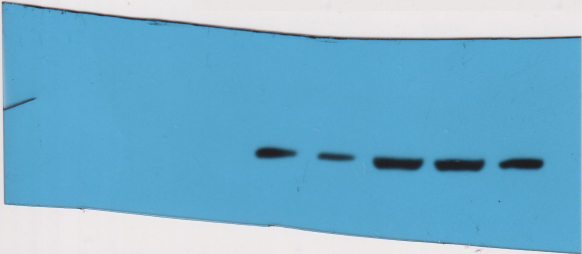

Supplement: Supplementary file 1 [file Data_Sheet_1.ZIP › Original Source Data/Figure 4/Figure 4A/MyD88.pdf]

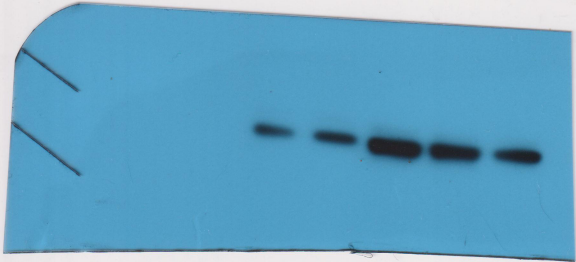

Supplement: Supplementary file 1 [file Data_Sheet_1.ZIP › Original Source Data/Figure 4/Figure 4A/NLRP3.pdf]

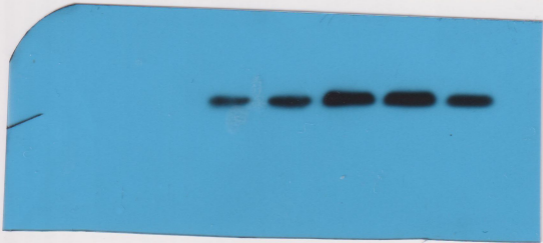

Supplement: Supplementary file 1 [file Data_Sheet_1.ZIP › Original Source Data/Figure 4/Figure 4A/p-p65.pdf]

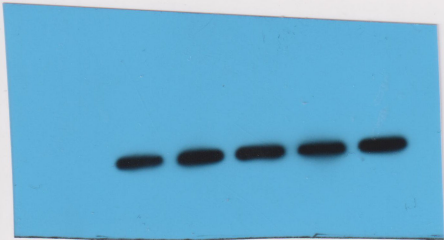

Supplement: Supplementary file 1 [file Data_Sheet_1.ZIP › Original Source Data/Figure 4/Figure 4A/p65.pdf]

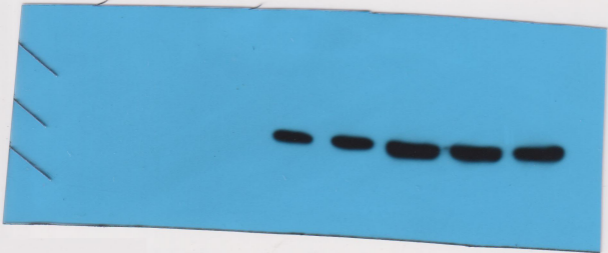

Supplement: Supplementary file 1 [file Data_Sheet_1.ZIP › Original Source Data/Figure 4/Figure 4A/TLR4.pdf]

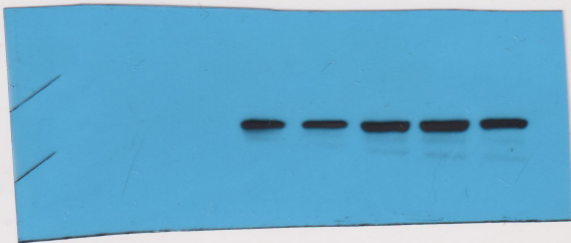

Supplement: Supplementary file 1 [file Data_Sheet_1.ZIP › Original Source Data/Figure 5/Figure 5A/Bax.pdf]

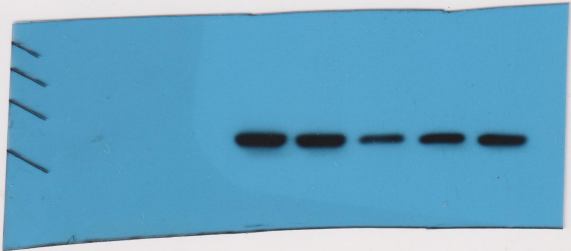

Supplement: Supplementary file 1 [file Data_Sheet_1.ZIP › Original Source Data/Figure 5/Figure 5A/Bcl-2.pdf]

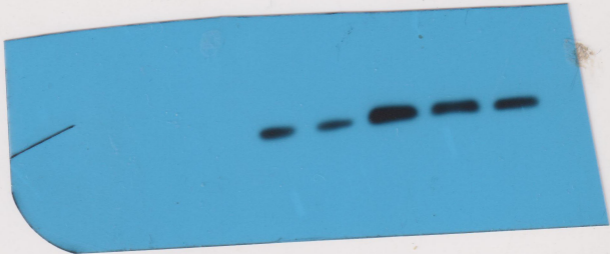

Supplement: Supplementary file 1 [file Data_Sheet_1.ZIP › Original Source Data/Figure 5/Figure 5A/Cleaved caspase-3.pdf]

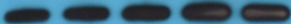

Supplement: Supplementary file 1 [file Data_Sheet_1.ZIP › Original Source Data/Figure 5/Figure 5A/GAPDH.pdf]

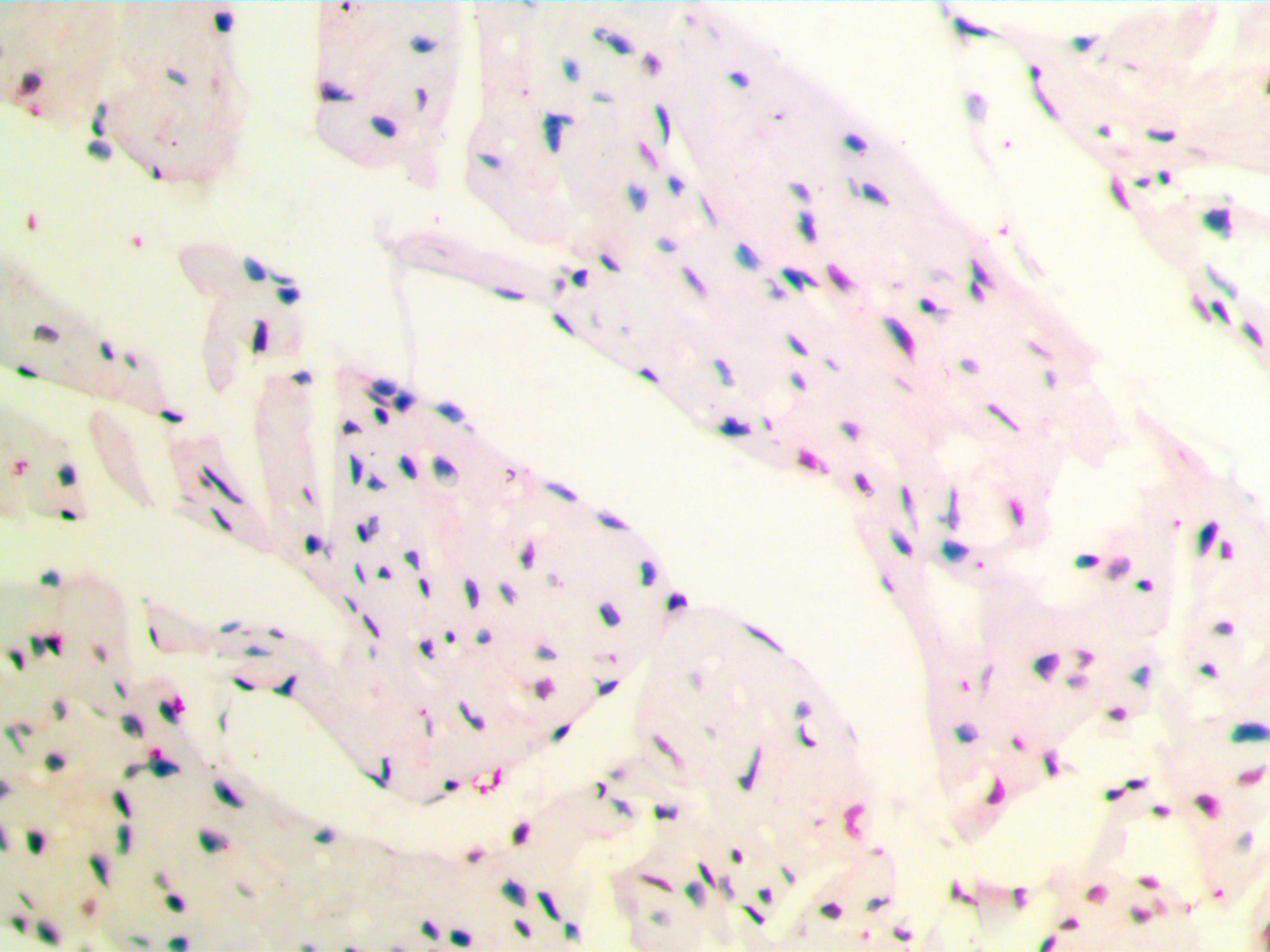

Supplement: Supplementary file 1 [file Data_Sheet_1.ZIP › Original Source Data/Figure 5/Figure 5F/Control.pdf]

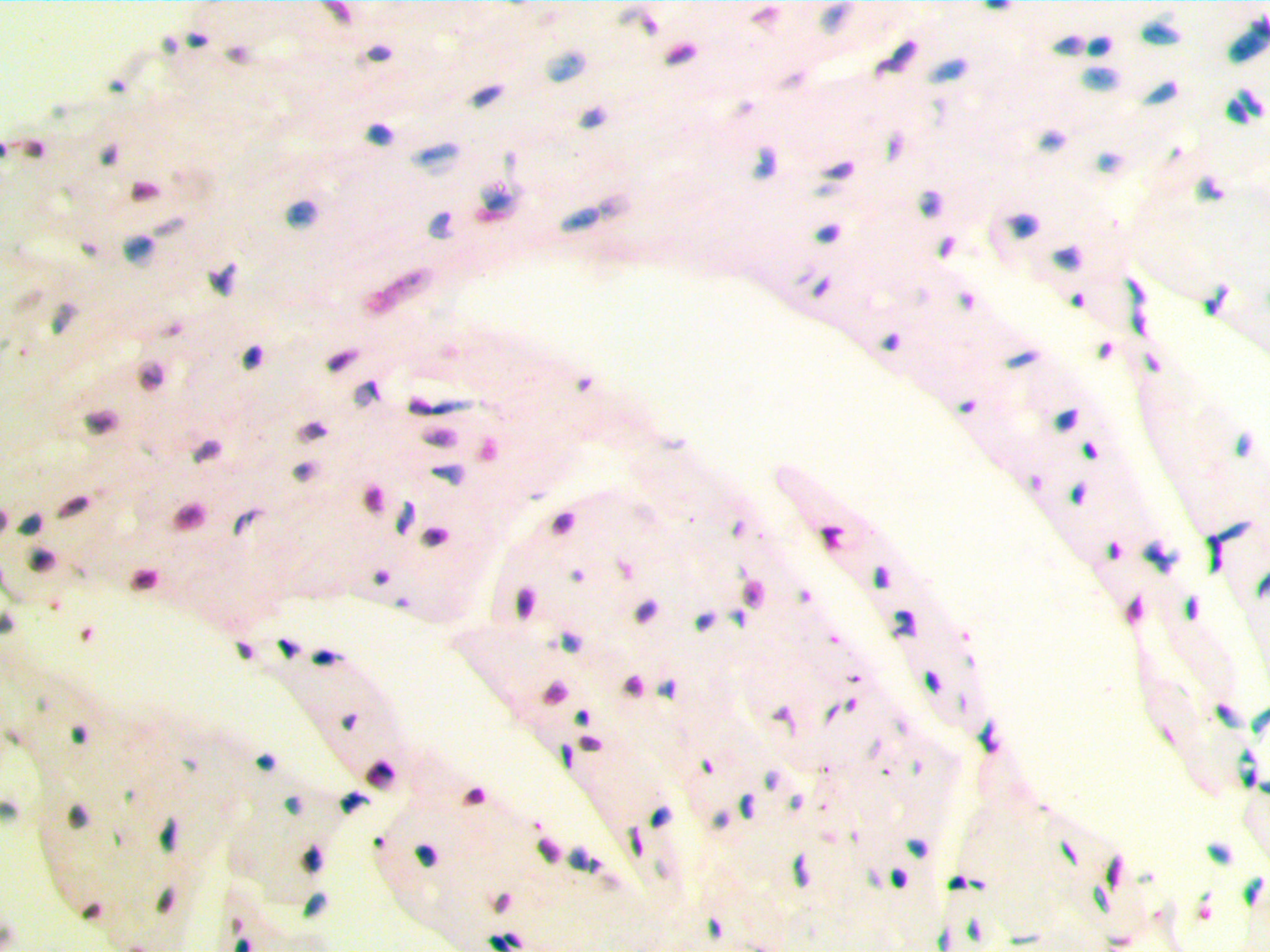

Supplement: Supplementary file 1 [file Data_Sheet_1.ZIP › Original Source Data/Figure 5/Figure 5F/IR + sh-NC.pdf]

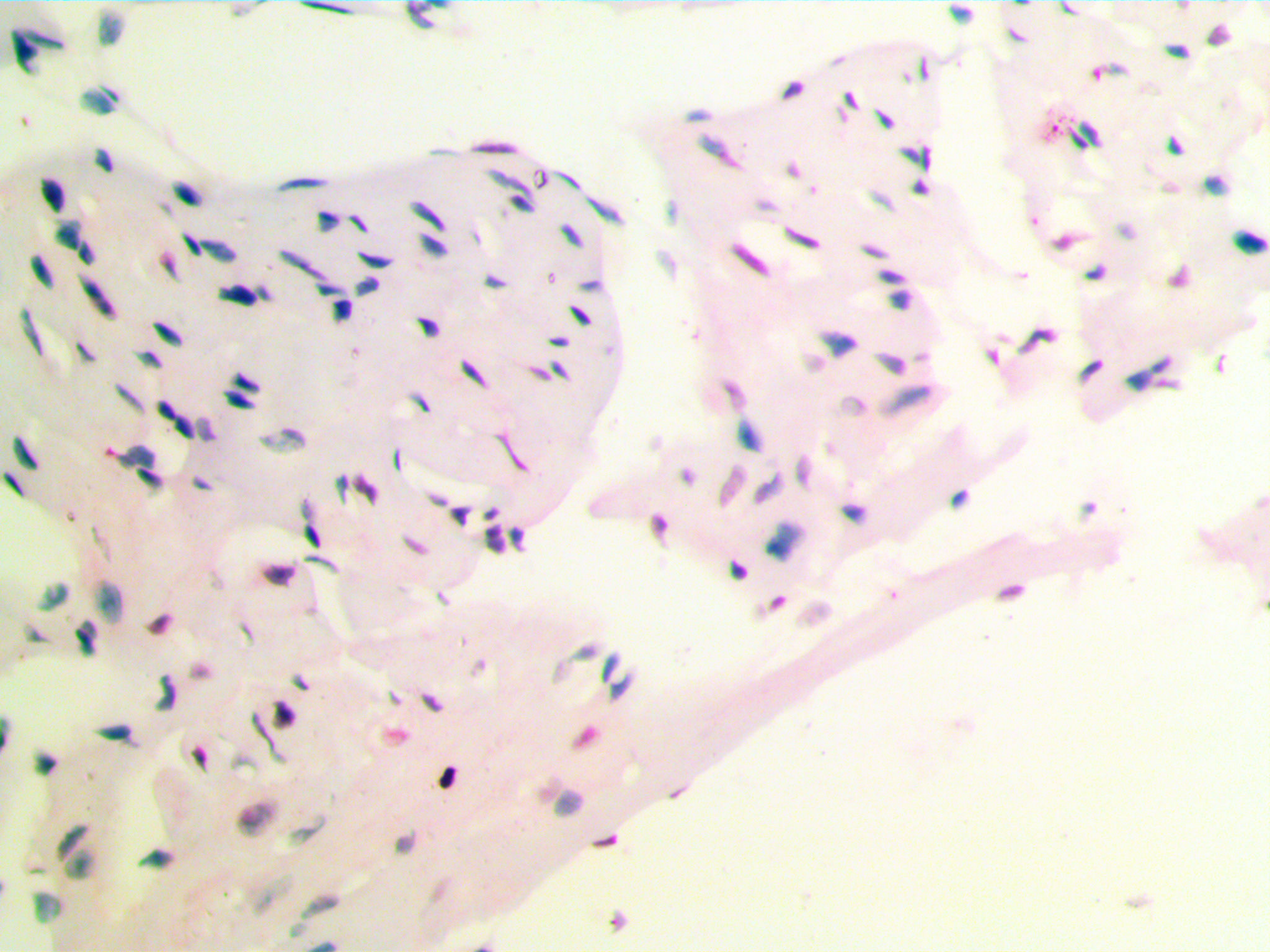

Supplement: Supplementary file 1 [file Data_Sheet_1.ZIP › Original Source Data/Figure 5/Figure 5F/IR + sh-PVT1.pdf]

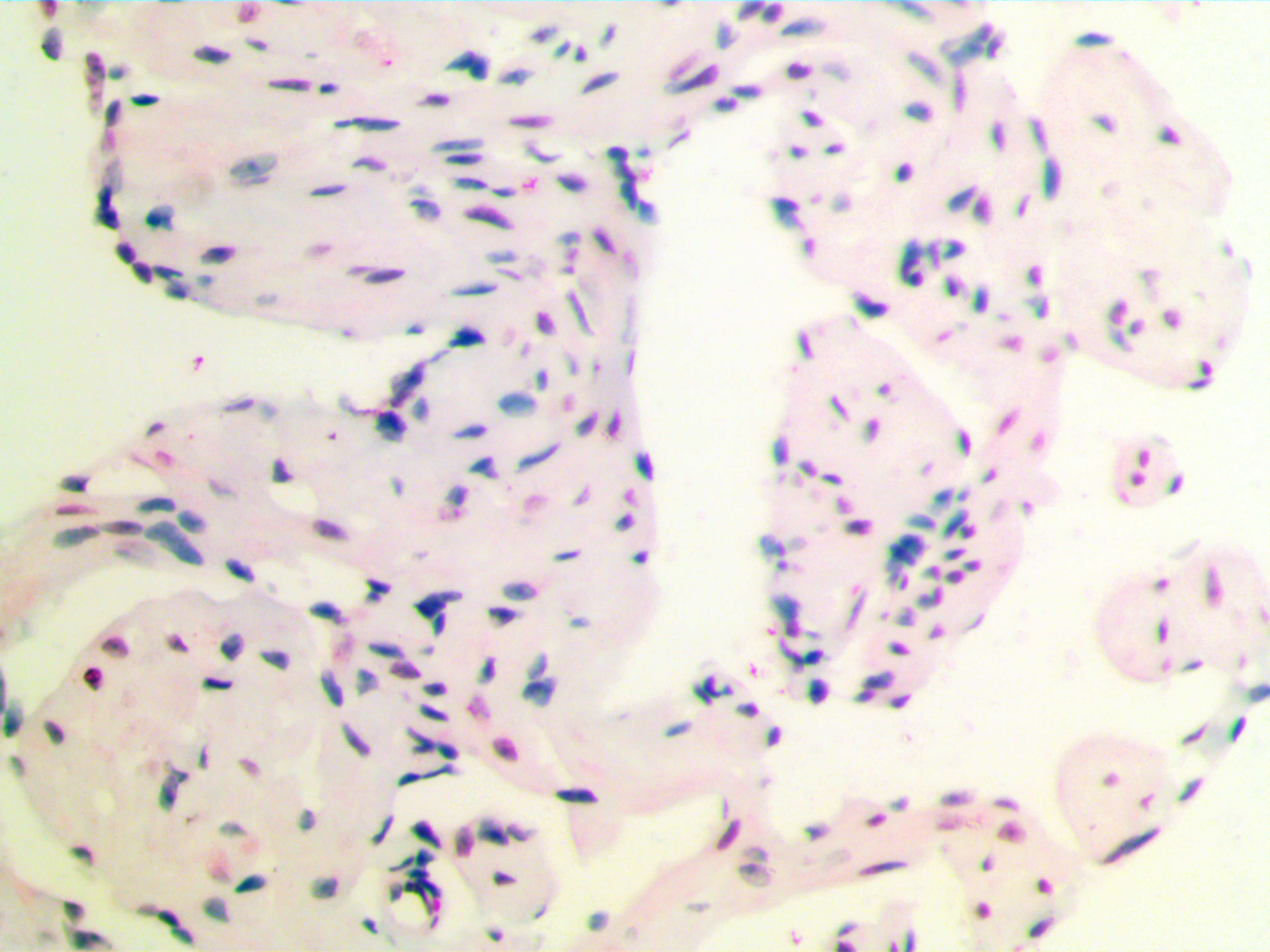

Supplement: Supplementary file 1 [file Data_Sheet_1.ZIP › Original Source Data/Figure 5/Figure 5F/IR.pdf]

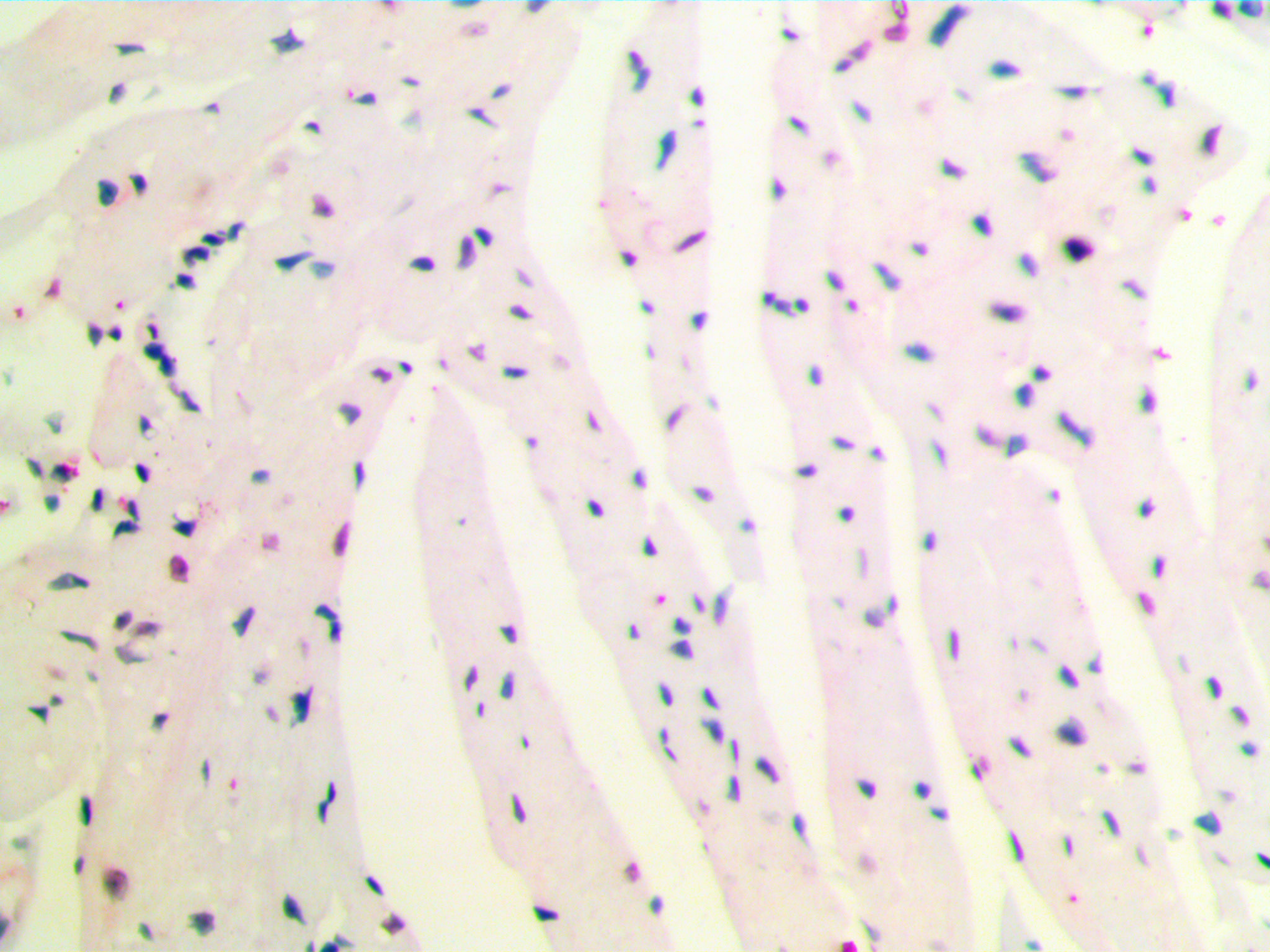

Supplement: Supplementary file 1 [file Data_Sheet_1.ZIP › Original Source Data/Figure 5/Figure 5F/Sham.pdf]

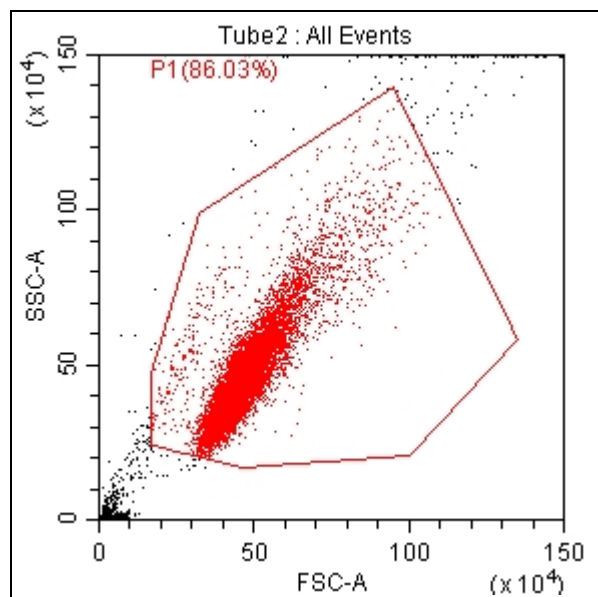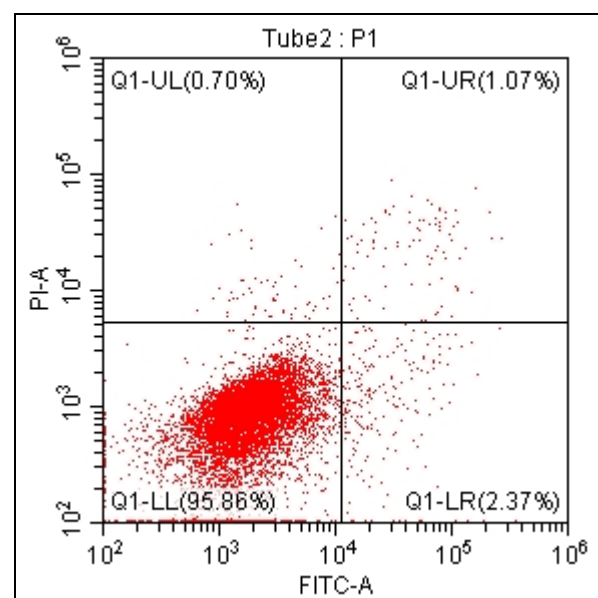

Supplement: Supplementary file 1 [file Data_Sheet_1.ZIP › Original Source Data/Figure 6/Figure 6D/Control.pdf]

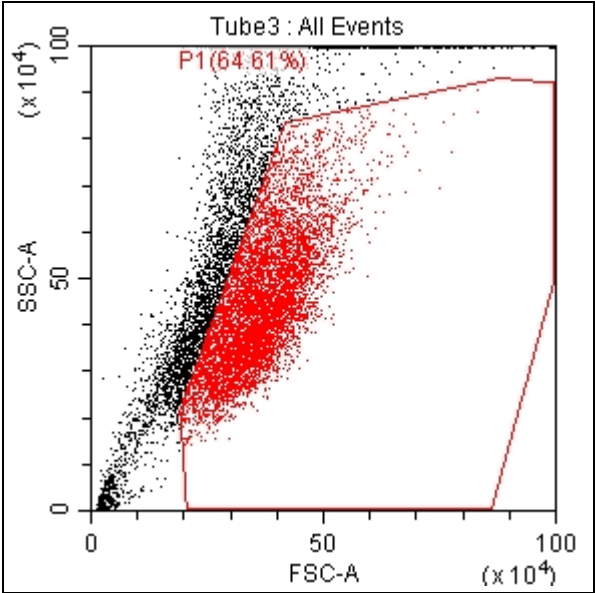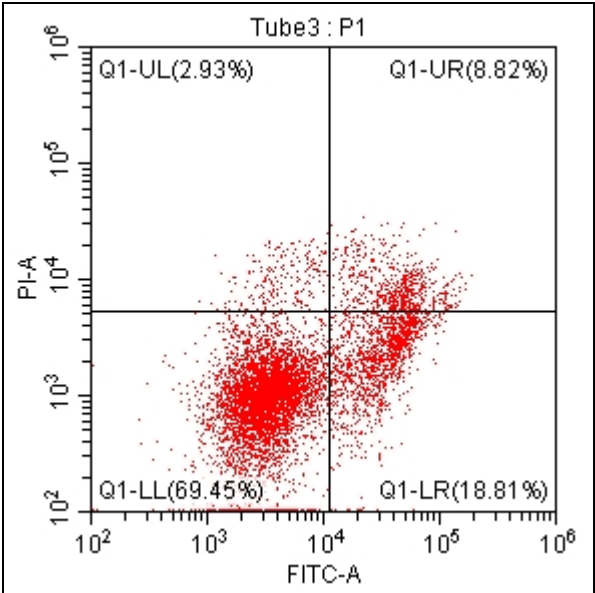

Supplement: Supplementary file 1 [file Data_Sheet_1.ZIP › Original Source Data/Figure 6/Figure 6D/HR+sh-NC.pdf]

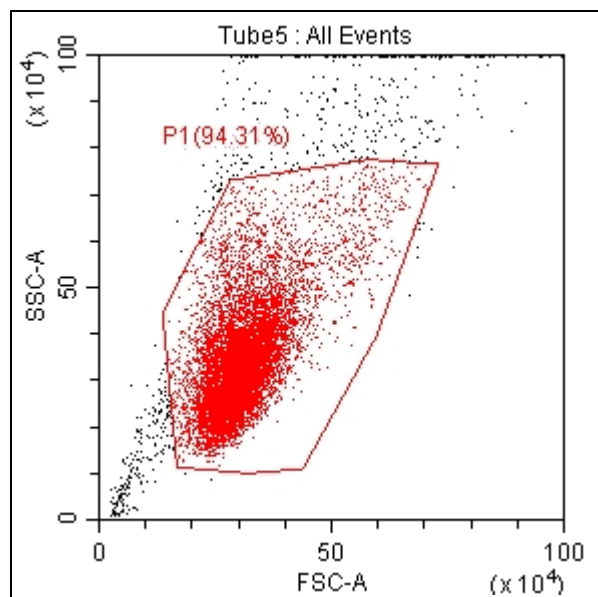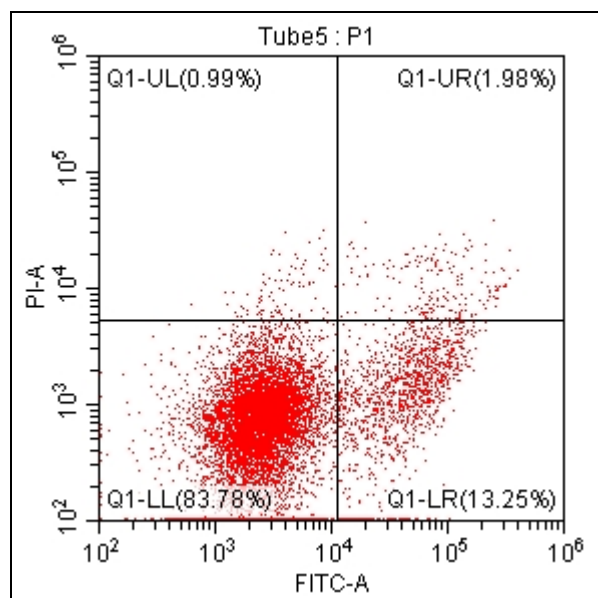

Supplement: Supplementary file 1 [file Data_Sheet_1.ZIP › Original Source Data/Figure 6/Figure 6D/HR+sh-PVT1.pdf]

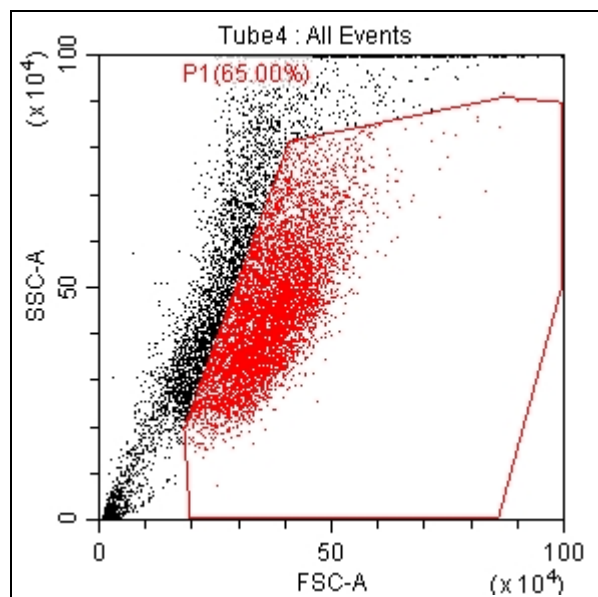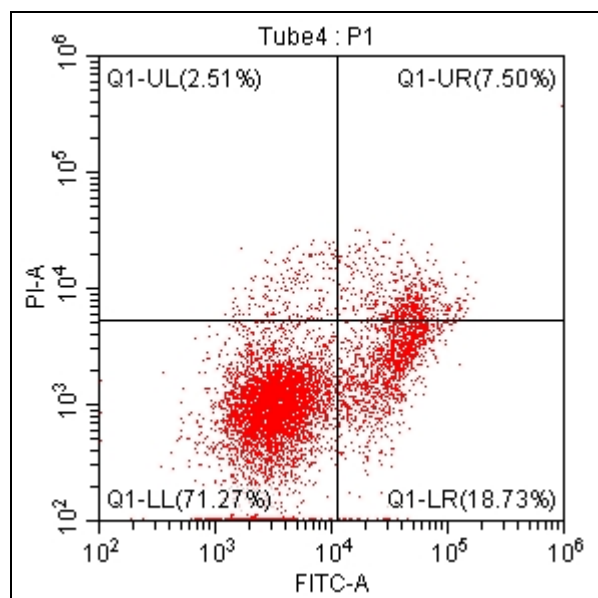

Supplement: Supplementary file 1 [file Data_Sheet_1.ZIP › Original Source Data/Figure 6/Figure 6D/HR.pdf]

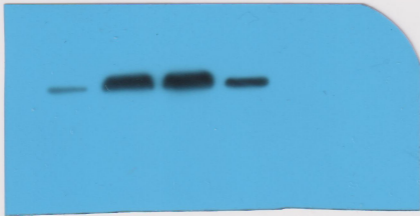

Supplement: Supplementary file 1 [file Data_Sheet_1.ZIP › Original Source Data/Figure 7/Figure 7B/Cleaved caspase1,p20.pdf]

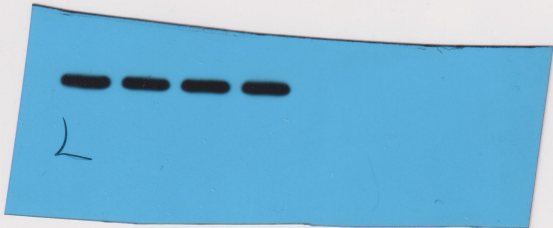

Supplement: Supplementary file 1 [file Data_Sheet_1.ZIP › Original Source Data/Figure 7/Figure 7B/GAPDH.pdf]

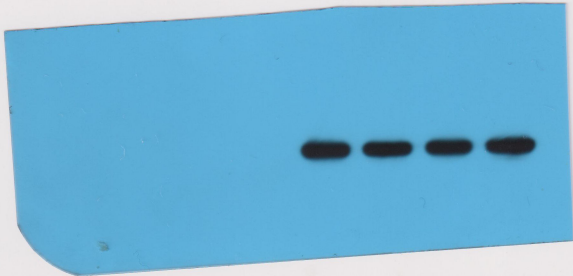

Supplement: Supplementary file 1 [file Data_Sheet_1.ZIP › Original Source Data/Figure 7/Figure 7B/GSDMD-FL.pdf]

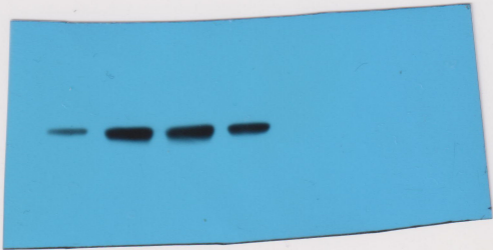

Supplement: Supplementary file 1 [file Data_Sheet_1.ZIP › Original Source Data/Figure 7/Figure 7B/GSDMD-N.pdf]

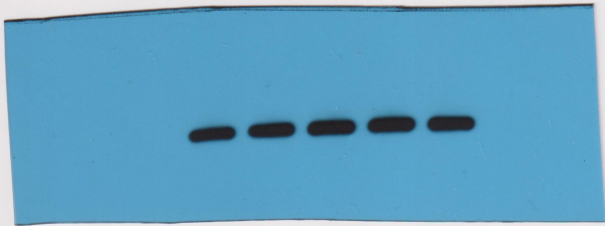

Supplement: Supplementary file 1 [file Data_Sheet_1.ZIP › Original Source Data/Figure 8/Figure 8D/GAPDH.pdf]

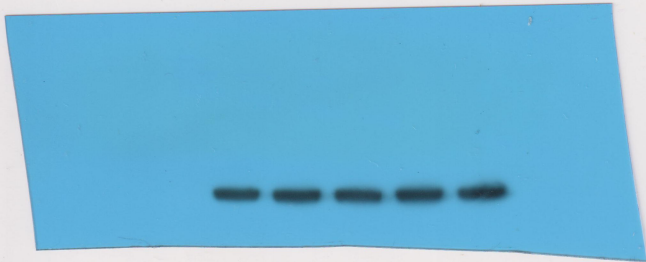

Supplement: Supplementary file 1 [file Data_Sheet_1.ZIP › Original Source Data/Figure 8/Figure 8D/GSDMD-FL.pdf]

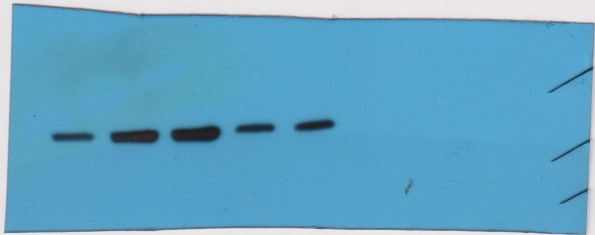

Supplement: Supplementary file 1 [file Data_Sheet_1.ZIP › Original Source Data/Figure 8/Figure 8D/GSDMD-N.pdf]
