# Supplementary material for: LncRNA PVT1 Knockdown Ameliorates Myocardial Ischemia Reperfusion Damage via Suppressing Gasdermin D-Mediated Pyroptosis in Cardiomyocytes
Source: Front Cardiovasc Med. 2021 Sep 14;8:747802. doi: 10.3389/fcvm.2021.747802 (PMC8476808; doi:10.3389/fcvm.2021.747802)

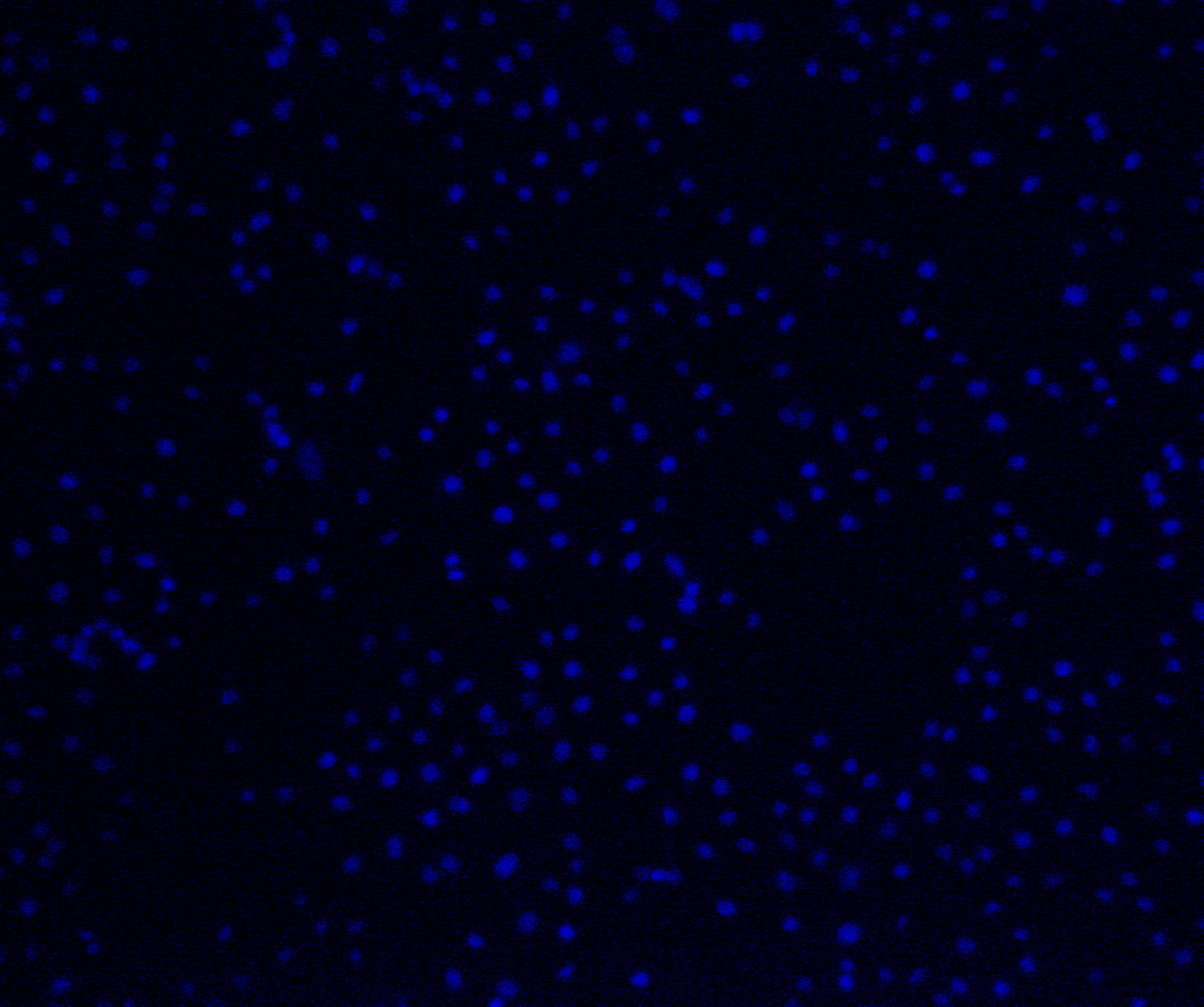

Supplement: Supplementary file 1 [file HR+sh-PVT1-DAPI.pdf › Original Source Data/Figure 7/Figure 7G/HR+sh-PVT1-DAPI.pdf]
